# Microbial Communities and Bioactive Compounds in Marine Sponges of the Family Irciniidae—A Review

**DOI:** 10.3390/md12105089

**Published:** 2014-09-30

**Authors:** Cristiane C. P. Hardoim, Rodrigo Costa

**Affiliations:** Microbial Ecology and Evolution Research Group, Centre of Marine Sciences (CCMar), University of Algarve (UAlg), Gambelas, 8005-139 Faro, Portugal; E-Mail: cristianehardoim@gmail.com

**Keywords:** host-microbe interactions, microbial diversity, polyketide synthases, secondary metabolites, symbiosis

## Abstract

Marine sponges harbour complex microbial communities of ecological and biotechnological importance. Here, we propose the application of the widespread sponge family Irciniidae as an appropriate model in microbiology and biochemistry research. Half a gram of one Irciniidae specimen hosts hundreds of bacterial species—the vast majority of which are difficult to cultivate—and dozens of fungal and archaeal species. The structure of these symbiont assemblages is shaped by the sponge host and is highly stable over space and time. Two types of quorum-sensing molecules have been detected in these animals, hinting at microbe-microbe and host-microbe signalling being important processes governing the dynamics of the Irciniidae holobiont. Irciniids are vulnerable to disease outbreaks, and concerns have emerged about their conservation in a changing climate. They are nevertheless amenable to mariculture and laboratory maintenance, being attractive targets for metabolite harvesting and experimental biology endeavours. Several bioactive terpenoids and polyketides have been retrieved from Irciniidae sponges, but the actual producer (host or symbiont) of these compounds has rarely been clarified. To tackle this, and further pertinent questions concerning the functioning, resilience and physiology of these organisms, truly multi-layered approaches integrating cutting-edge microbiology, biochemistry, genetics and zoology research are needed.

## 1. Introduction

The phylum Porifera (sponges) represents the oldest extant metazoan lineage on Earth, with fossil records dating to around 580 million years ago [1,2]. The ancestry of marine sponges might even be older than previously thought, as chemical records found for demosponges place the origin of these animals back to more than 635 million years ago at the transition between the Ediacaran (Neoproterozoic) and the Cambrian (Paleozoic) periods [3]. Sponges are sessile filter-feeders, with capability to filter thousands of litres of water per day [4,5,6]. They can be found in almost all of the 232 marine ecoregions of the World [7]. Eleven such ecoregions encompass 201–461 species, and are thus considered hot spots of marine sponge diversity [2,7] (Table 1). These animals are restricted to aquatic habitats and divided into four classes: Demospongiae, Hexactinellida, Calcarea and Homoscleromorpha, which are further distributed into 25 orders, 128 families, and 680 genera. The class Demospongiae is by far the richest and most widespread, containing 83% of the 8553 formally recognized species described so far [2].

**Table 1:** Hot spots of marine sponge biodiversity.

Realms ^1^	Provinces	Ecoregions	Number of Species ^2^
Tropical Atlantic	Tropical North-western Atlantic	Eastern Caribbean	208
Tropical Atlantic	Tropical North-western Atlantic	Greater Antilles	275
Temperate Northern Atlantic	Northern European Seas	Celtic Seas	295
Temperate Northern Atlantic	Lusitanian	South European Atlantic Shelf	231
Temperate Northern Atlantic	Lusitanian	Azores, Canaries, Madeira	458
Temperate Northern Atlantic	Mediterranean Sea	Western Mediterranean	461
Western Indo-Pacific	West and South Indian Shelf	South India and Sri Lanka	211
Temperate Northern Pacific	Warn Temperate Northwest Atlantic	Central Kuroshio Current	377
Central Indo-Pacific	Tropical South-western Pacific	New Caledonia	256
Temperate Australasia	East Central Australian Shelf	Manning-Hawkesbury	278
Temperate Australasia	Southeast Australian Shelf	Bassian	375

^1^ “Realms”, “Provinces” and “Ecoregions” are defined according to Spalding and colleagues [7]; ^2^ The number of sponge species per Ecoregion [7] was obtained from van Soest and colleagues [2].

**Figure 1:**
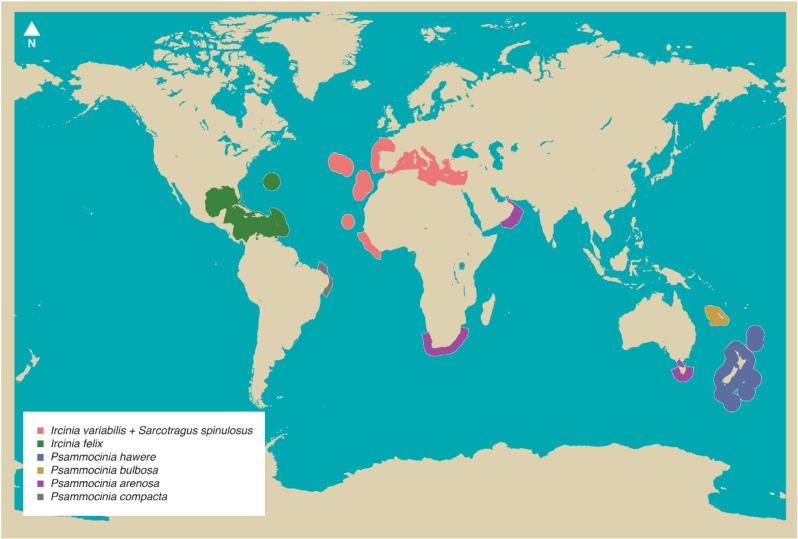
Geographical distribution of exemplarily Irciniidae species. The map was drawn using data available at the World Porifera Database; of note is the wide longitudinal occurrence of the genus *Psammocinia*.

Although prokaryotic microorganisms are the most important component of their diet, sponges are known to form symbiotic associations with abundant and diverse bacteria and, in some cases, up to 38% of sponge wet weight consists of bacterial cells [4,5,6]. So far, 10 recognized bacterial phyla and 18 candidate bacterial phyla have been detected in marine sponges [5,6,8]. Several lineages within these phyla are involved in the production of secondary metabolites [9], which may enhance host defence mechanisms against predators and invading pathogens. Besides bacteria, archaea and fungi are consistently found as constituent members of the marine sponge microbiome, and specific host-related functions have been proposed or reported for these groups [6,9]. In the light of the enormous richness of marine sponges across the globe, their contribution as the most prolific sources of marine bioactive compounds and the actual participation of their microbial symbionts in secondary metabolite biosynthesis, it is reasonable to posit that marine sponge microbiomes constitute as-yet uncharted, extremely fertile reservoirs of genetic and metabolic novelties. In this context, the present review covers microbial community structure, diversity and bioactivities reported for marine sponges of the family Irciniidae (Demospongiae, Dictyoceratida), known for its wide geographical distribution (Figure 1), chemical complexity and distinct microbiota. In addition, we make use of the Irciniidae family as a model taxon to revisit our knowledge of marine sponge (host and symbiont) cell culturing, metagenomics-based gene discovery, experimental biology, cell-cell signalling and disease. We follow the concept of symbiosis as originally proposed by Anton de Bary (see Taylor *et al.* [6]) to describe two or more organisms living in close physical association over time. This definition does not imply any sort of mutual benefit between hosts and symbionts.

## 2. The Irciniidae Family as a Model Taxon in Sponge Microbiology Research

**Figure 2:**
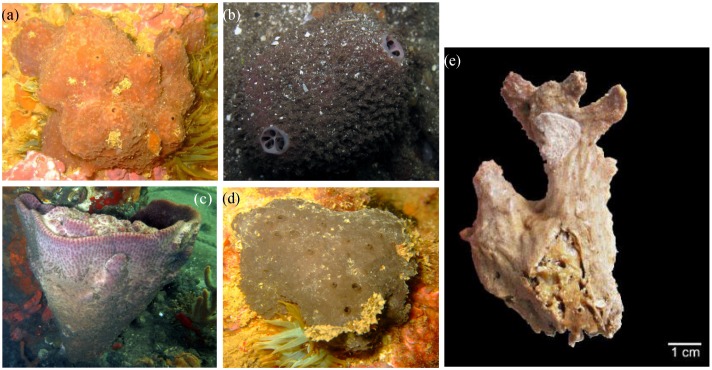
*In situ* pictures of *Ircinia variabilis* (**a**); *Ircinia felix* (**b**); *Ircinia campana* (**c**); *Sarcotragus spinosulus* (**d**) and of a fixed specimen of *Psammocinia compacta* (**e**); Photos courtesy: Francisco R. Pires (**a**–**d**); Panel (**e**) is reproduced with permission from Guilherme Muricy (Department of Invertebrates, Federal University of Rio de Janeiro, Brazil).

With 8553 catalogued and 17,000 estimated species, comprehensively describing microbial diversity, metabolism and functioning in marine sponges is a complex task. Determining how these features relate to host species, habitat, depth and environmental gradients therefore constitutes a true challenge. Given this, the need to identify and develop model hosts in sponge microbiology research has been discussed by specialists at recent meetings such as the 1st International Symposium on Sponge Microbiology [10] and the Lower Invertebrates Symbiosis with Microorganisms Workshop held in Eilat, Israel in 2012. In the past 15 years, intense molecular microbiology research performed on, for example, *Aplysina aerophoba* from the Mediterranean Sea [11,12,13,14,15], *Rhopaloeides odorabile* from the Great Barrier Reef (Australia) [16,17,18,19,20] and *Ircinia* spp. from the Caribbean [21,22,23,24,25] have naturally turned these organisms into model sponge hosts. Strategic and long-term research on key species will most likely improve our understanding of sponge-microbe interactions in a more mechanistic fashion, allowing us to extract pertinent information from a core of relevant hosts that could represent the whole. In this regard, a likely rewarding approach to the choice of model organisms is looking for taxonomic ranks above the species level. This would widen the biodiversity spectrum of the target animals, expanding the geographical, habitat and niche breadths as well as the phenotypic and genotypic plasticity ranges under study. Such a strategy could thus facilitate the assessment of microbiome diversity and function as a response to environmental and host-related factors within a well-contextualized phylogenetic framework. The family Irciniidae is composed only of marine species divided into three genera, namely *Ircinia*, *Sarcotragus* and *Psammocinia*, embracing 75, 11 and 25 accepted species, respectively [26] (see Figure 2 for pictures of some Irciniidae species). Most species have been registered mainly from tropical to temperate regions [27] (see Figure 1 for the geographical distribution of exemplarily species) and inhabit the epipelagic layer (*i.e.*, photic zone where there is enough light for photosynthesis) from zero to 60 m depths. However, a few specimens have been collected from the mesopelagic layer at about 365 m [28]. Irciniidae species exhibit a wide variety of shapes (for a technical description, see [27]) and lack authentic spicules, the archetypical mineral skeleton-forming structures in the majority of demosponges [29]. Instead, fine collagenous and terminally enlarged spongin filaments are typical features of this family. These constitute the organic fibre skeleton, making the sponges very difficult to tear. Several reasons suggest the Irciniidae as a suitable model taxon for microbiology and biotechnology research, as follows:

1. The existence of several sympatric species within the family allows testing the host species-specific hypothesis of microbiome composition in sponges using a solid phylogenetic background;
2. The wide geographical distribution of some species permits testing hypotheses of symbiont/metabolite maintenance across biogeographical gradients;
3. Irciniidae species are chemically rich, a feature that might relate with microbial diversity and suggests biotechnological potential;
4. Irciniidae species have been reported as high microbial abundance sponges. This suggests high metabolic activity and/or selective enrichment of their symbionts, which most likely play essential roles for host fitness and survival;
5. Bacterial species from at least 15 phyla have been found to be enriched in Irciniidae hosts when compared with surrounding seawater. This distinct microbiome composition is indicative of close host-symbiont relationships;
6. The order Dictyoceratida consists of four families—including Irciniidae—collectively known as “keratose” sponges because their skeleton is made of a complex matrix of protein fibres instead of mineral elements (*i.e.*, true spicules), making these families interesting representatives of a peculiar life strategy within marine sponges.

**Figure 3:**
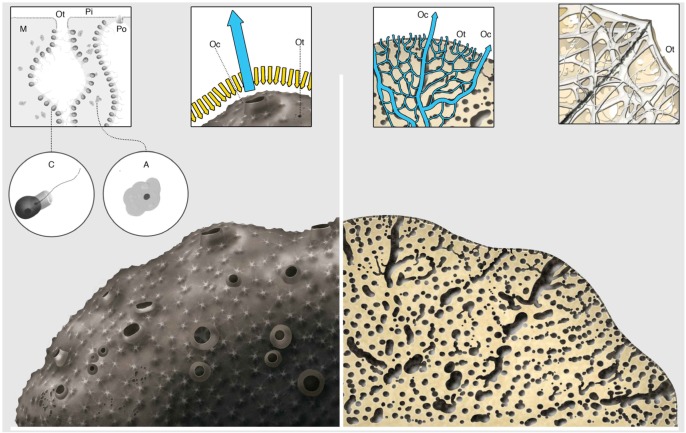
Schematic overview of a demosponge from the family Irciniidae. Mesohyl (M), ostium (Ot), pinacoderm (Pi), porocyte (Po), choanocyte (C); archeocyte (A); and osculum (Oc); arrows indicate the water flow within the sponge body.

In the following sections, we provide a historical perspective on the marine sponge microbiome, with focus on irciniid hosts (see Figure 3 for a schematic representation of a specimen from the family). It is not the scope of this review to describe all bioactive compounds retrieved from Irciniidae species. Instead, an extensive list of metabolites documented in these sponges is given as Supplementary Information, with notes of a possible microbial origin of the described compounds.

## 3. Microbial Diversity and Bioactivities in the Family Irciniidae

Microbial diversity surveys have focused primarily on Caribbean species belonging to the genus *Ircinia* [21,22,23,24,30,31,32,33] and on *Ircinia* and *Sarcotragus* species from the eastern Atlantic Ocean and Mediterranean Sea [34,35,36,37,38,39,40]. However, no direct comparison between prokaryotic communities from irciniids inhabiting these regions has been performed. Previous studies classified members of the Irciniidae family as high microbial abundance (HMA) sponges [31,38], highlighting their capability of hosting highly dense microbiomes, ranging from 10^8^–10^1^^0^ prokaryotic cells·g^−1^ of sponge (fresh weight). Although several attempts have been made to isolate secondary metabolites from these sponges (see Supplementary Information), little attention was paid to determine the actual producer (*i.e.*, host or symbiont) of such compounds.

### 3.1. Early Microbiology Studies

First insights into the microbial abundance and morphological diversity in sponges were obtained by electron microscopy—especially transmission electron microscopy (TEM) [4,41], still an important tool in several studies [12,42,43,44,45]. In the early 1970s, Sara [46] showed that *I. variabilis* was populated by the cyanobacteria *Aphanocapsa feldmanni* and *A. raspaigellae*. The former was detected in the cortical mesohyl—the intercellular matrix of sponges—and inside sponge cells, while the latter was found only in the mesohyl, surrounded by a lacunar space. Both species reproduced outside the host’s cells. A true cooperative relationship was suggested between host and symbionts, whereby *Aphanocapsa* spp. supplied the sponge with organic material originated from photosynthesis, protected the host from excessive illumination and could use the nitrogenous compounds eliminated by the sponge, whereas *I. variabilis* offered shelter and protection for the Cyanobacteria species [46]. Using scanning and transmission electron microscopy, Wilkinson [47,48] revealed higher abundance of heterotrophic bacteria in the dense mesohyl of *I. wistarii* from the Great Barrier Reef (Australia) when compared with surrounding seawater (SSW). These symbionts concentrated around the inhalant canals of the sponge. Although pioneering TEM analyses enabled first assessments of the abundance and morphology of bacteria in sponges, the taxonomic diversity and composition of these symbionts remained enigmatic until the first culture-dependent and -independent inventories of sponge-associated bacteria were created. These were ultimately responsible for the enormous increase in knowledge regarding sponge microbiome diversity in the last 35 years.

### 3.2. Bacteria

#### 3.2.1. Diversity

##### 3.2.1.1. Culture-Dependent Approaches

Wilkinson [48] isolated 87 bacterial colonies from *I. wistarii* and performed 76 tests measuring morphology, physiology and metabolic capabilities. Fifty-nine isolates were grouped into six well-defined clusters, whereas 28 isolates were too ambiguous to be assigned to any clade. Surprisingly, these isolates were able to metabolize a wide range of compounds and, for the first time, comprehensive information on the physiology of sponge-associated bacteria was provided. During the subsequent 25 years, no attempt was made to cultivate symbionts from Irciniidae species, whereas several other sponge hosts have been approached in this manner. These studies usually reported on Alpha- and Gamma classes of Proteobacteria as the prevailing members of the culturable sponge-associated microbiota [42,43,49]. Likewise, the culturable assemblage of *Ircinia* sp. (St. Giovanni, Croatia) was later found to be dominated by Alpha and Gammaproteobacteria [50]. In another survey, Esteves and colleagues [37] isolated more than 270 bacterial strains from *S. spinosulus* and *I. variabilis* on marine agar. These were classified into 17 genera, and 10 putative new species were detected. In accordance with several cultivation-dependent studies performed with other hosts [42,51,52,53], the bacterial genera *Pseudovibrio*, *Ruegeria* and *Vibrio* (Proteobacteria) prevailed among the retrieved symbionts [37]. Using six Actinobacteria-specific media, Tabares and colleagues [54] isolated only one single strain, *Arthrobacter* sp. BA51, from *I. felix*. The main advantage of bacterial cultivation is that this allows the determination of the putative functions of these symbionts. For instance, some studies [37,50] have tested bacterial cultures from Irciniidae specimens for *in vitro* antimicrobial activity (see Section 3.2.2), an approach widely used in sponge microbiology. However, the biases inherent in culture-dependent methods, such as <1% of the total bacterial cells being recovered [42,55], with most of the isolates belonging to the Proteobacteria [6,43,56], have favoured the use of cultivation-independent techniques in the characterization of sponge-associated bacterial communities.

##### 3.2.1.2. Cultivation-Independent Approaches

**Figure 4:**
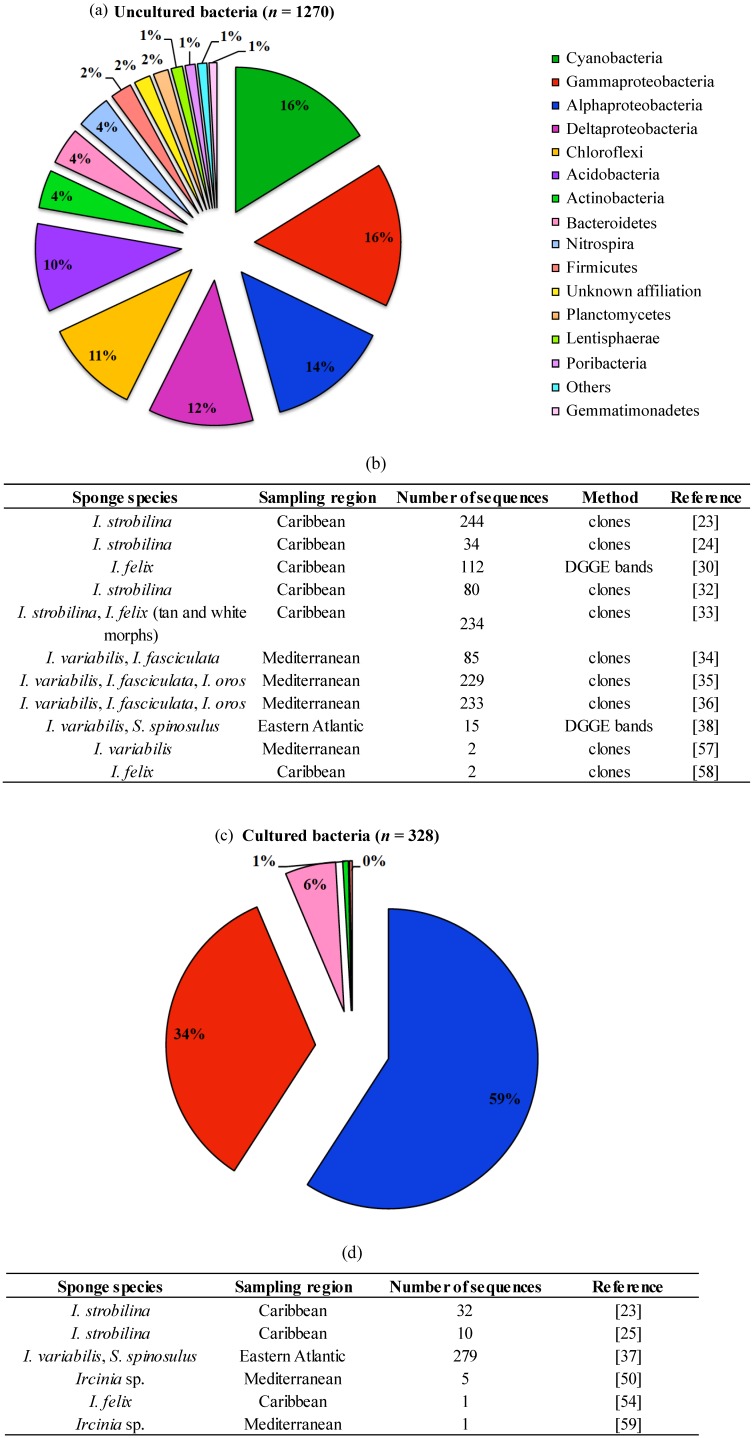
Phylum-level classification of bacteria detected in Irciniidae sponges by cultivation-independent (**a**) and cultivation-dependent (**c**) 16S rRNA gene surveys, and their respective metadata (**b**,**d**); the analysis covers all sequences available at NCBI until December 2013, with the exception of 454-pyrosequencing sequence reads. The phylum Proteobacteria is represented in (**a**) and (**c**) by the classes Alpha, Gamma and Deltaproteobacteria. The total number of sequences analysed in (**a**) and (**c**) is given in brackets.

The application of DNA-based, molecular approaches to the marine sponge microbiome, relying mainly on the sequencing or fingerprinting of 16S rRNA genes, has illuminated our view of the bacterial community structure and diversity in these organisms by circumventing the well-known limitations of culturing techniques. Indeed, a meta-analysis performed with 16S rRNA gene sequences derived from irciniid sponges via cultivation-dependent and -independent methods reveals that the latter strategy uncovers much higher bacterial diversity from these hosts than the former (Figure 4). Below, we first address pioneering fingerprinting/cloning-and-sequencing studies, and then recent surveys that use next generation sequencing to inspect bacterial community structure in marine sponges with cultivation-independent methods.

Using a Cyanobacteria-specific primer pair, Usher and colleagues [57] detected, by polymerase chain reaction-denaturing gradient gel electrophoresis (PCR-DGGE), two closely related species of *Synechococcus* in *I. variabilis*. PCR-DGGE fingerprinting and clone-and-sequencing of 16S rRNA gene fragments using universal bacterial primers were later applied to determine the structure of bacterial communities associated with *I. felix* and *I. strobilina* from Key Largo, Florida, USA [23,30,31]; *I. strobilina* from Sweetings Cay, Bahamas [32]; and the Mediterranean *I. variabilis*, *I. fasciculata* (currently accepted taxon: *Sarcotragus fasciculatus*. For the sake of clarity, in this review we will keep the source name *Ircinia fasciculata* whenever referring to previous studies) and *I*. *oros* [34]. These studies revealed Acidobacteria, Actinobacteria, Chloroflexi, Cyanobacteria, Gemmatimonadetes, Nitrospira and Proteobacteria (Alpha, Delta and Gamma classes) as the prevailing bacterial phyla in *Ircinia* spp. It was also demonstrated that irciniids harboured a distinct microbiome compared to SSW [23,30,31,32,33,34]. Erwin and colleagues [34] showed that 38 out of 56 sequences fell within “sponge-specific” or sponge- and coral-specific clusters. The number of 99% operational taxonomic units (OTUs, here defined at 99% gene sequence similarity) found in *I. fasciculata*, *I. oros* and *I. variabilis* was 29, 33 and 34, respectively. Only four OTUs were registered in all three *Ircinia* species, revealing that each sponge host harboured its own unique bacterial community. Erwin and colleagues [35] further monitored bacterial community structures in the aforementioned species and SSW once every season for 18 months. Terminal restriction fragment length polymorphism (T-RFLP) analysis revealed that the bacterial communities within each host were highly stable over time. A 16S rRNA gene cloning-and-sequencing procedure using universal bacterial primers was further employed, whereby the same sponge individuals were collected in winter and summer. This showed that >50% of the sponge symbionts were maintained across seasons with no significant differences in community structure. Seven out of eight OTUs were closely related to typical sponge-associated bacteria, of which *Candidatus* S. spongiarum (Cyanobacteria) was the most dominant followed by OTUs affiliated with Deltaproteobacteria, Acidobacteria, Gammaproteobacteria, Nitrospira and Cyanobacteria. Erwin and colleagues [36] also unravelled Cyanobacteria diversity and abundance in *I. fasciculata* and *I. variabilis* using several approaches. Cyanobacterial cells were found in the ectosome of both sponge species in dense populations*,* but *I. fasciculata* contained almost twice the level of chlorophyll *a* and higher abundance of glycogen granules than *I. variabilis.* *Candidatus* S. spongiarum was again dominant, being detected intercellularly in the mesohyl, actively reproducing, and seemed to interact with host cells. *Synechocystis* sp. was sporadically found in *I. fasciculata*, and it was neither in reproductive process nor in close association with host cells. These results corroborate the findings of Usher and colleagues [57], suggesting that cyanobacterial symbionts play an important role in photosynthesis within these sponges [36]. Hardoim and colleagues [38] determined the bacterial abundance, diversity and community composition in *S.* *spinosulus* and *I.* *variabilis*. Both sponge species were for the first time classified as HMA sponges based on epifluorescence microscopy analysis. PCR-DGGE profiles of *S. spinosulus* and *I. variabilis* were dissimilar, showing that each sponge species harboured its own bacterial community, strengthening the findings of Erwin and colleagues [34] on specific bacterial assemblages inhabiting sympatric Irciniidae species. Pita and colleagues [39] further examined the spatial stability of bacteria associated with *I. fasciculata*, *I. oros* and *I. variabilis* collected at six locations in the western Mediterranean Sea. T-RFLP analyses showed no reliable clustering of sponge symbiont communities according to their respective sampling sites, suggesting high stability of these assemblages across distances from 80–800 km. Pita and colleagues [33] also disclosed the spatial variability and host specificity of bacteria associated with *I. strobilina* and two colour morphs of *I. felix* collected at five islands representing different environmental conditions in the Bahamas. T-RFLP fingerprinting uncovered different bacterial communities from *I. strobilina* and *I. felix*. However, the same was not true for both colour morphs of *I. felix.* No significant correlations between bacterial community similarity and sampling sites were detected for both sponge species. The authors suggested that periodic horizontal (*i.e.*, seawater) acquisition together with continuous vertical transmission of symbionts (see below) might contribute to the specificity of bacterial communities within each host sponge [33].

**Figure 5:**
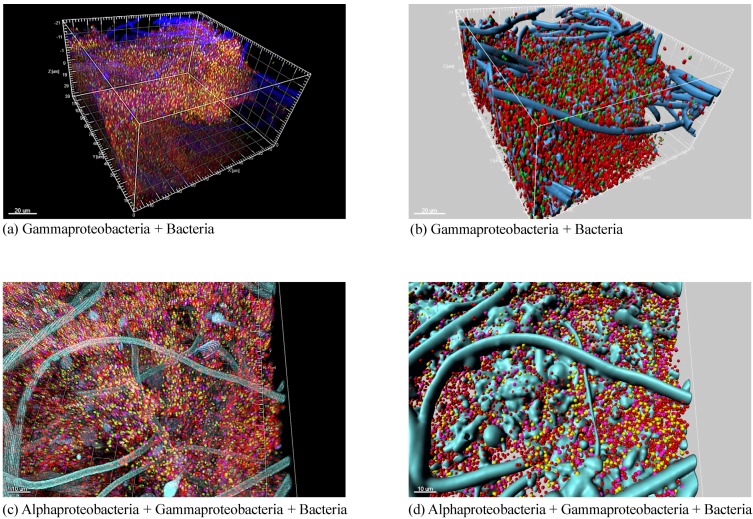
Confocal laser scanning microscopy images of fluorescent *in situ* hybridization (FISH-CLSM) stained bacteria in *S. spinosulus*; volume rendering images (left in each panel) and their corresponding 3D reconstructions (right in each panel) are shown for hybridizations with the Cy3-labeled universal bacterial probe (red cells) coupled to ALEXA488- or Cy5-labeled group-specific probes targeting the Alpha- and Gammaproteobacteria classes; Gammaproteobacteria cells appear as yellowish cells in the volume rendering images (**a**) and as green objects in the 3D reconstruction (**b**). For co-hybridizations including bacterial, alpha and gammaproteobacterial probes, the latter two groups are represented by yellowish and pink cells, respectively (**c**,**d**). Sponge background structure, vastly dominated by profuse spongin filaments, is shown overall in blue or cyan.

To overcome limitations common to PCR-DGGE and T-RFLP fingerprinting (*i.e.*, impossibility to identify the entire prokaryotic community) and cloning-and-sequencing procedures (*i.e.*, restrictions regarding library sizes), next-generation sequencing (NGS, e.g., 454-pyrosequencing) has been more often applied in recent years for the characterization of sponge symbiont diversity [20,60]. Using 454-pyrosequencing, 16 bacterial phyla and 1199 OTUs (95% gene similarity cut-off) were recovered from *I. ramosa* sampled from the Great Barrier Reef (Australia), whereas 30 bacterial phyla and 6800 OTUs were recovered from SSW [20]. With this technology, Schmitt and colleagues [14] uncovered 159, 179, 86 and 111 bacterial OTUs (97% cut-off) from *Ircinia* sp., *I. felix*, *I. variabilis* and *I. gigantea* (note by authors: non-valid taxon) specimens sampled from the Indian Pacific, Caribbean and Mediterranean Seas, and the Great Barrier Reef, respectively. Overall, the dominant bacterial phyla associated with *Ircinia* spp. were Acidobacteria, Chloroflexi, Poribacteria and Proteobacteria (Alpha and Gamma classes)*.* Both surveys suggested that indeed the bacterial community associated with marine sponges was species-specific [14,20]. Likewise, 454-pyrosequencing was employed in recent studies covering cultivation bias, host specificity and temporal dynamics of microbial communities associated with Irciniidae species in the north Atlantic. Hardoim and colleagues [61] addressed the effects of three methods of sample handling on bacterial community composition in *S. spinosulus* and *I. variabilis*. Using cultivation-independent methods, the bacteriome of *S. spinosulus* and *I. variabilis* was indeed found to be species-specific, deepening the observations made by means of PCR-DGGE profiling [38], whereas similar bacterial assemblages were detected in both sponge species via culturing, highlighting the bias associated with describing sponge microbiome diversity based solely on cultivation attempts. These authors also localized bacterial symbionts in *I. variabilis* and *S. spinosulus* by fluorescence *in situ* hybridization (FISH). They observed that bacterial cells were almost exclusively detected in-between sponge cells and ususally absent on the spongin fibers that form the skeleton of Irciniidae sponges (Figure 5). In addition, Hardoim and Costa [62] unravelled the temporal dynamics of the bacterial community associated with *S. spinosulus* over three sucessive years. PCR-DGGE and tag-pyrosequencing showed that the bacterial assemblage was stable over time, with the majority of dominant bands and OTUs detected in all years. *S. spinosulus* was dominated by eight bacterial phyla, of which only two presented different relative abundances over the years. Only 27 OTUs, distributed in nine bacterial phyla, were detected in all 12 sponge specimens. Interestingly, in spite of its low diversity at the phylotype level, this minimal core of symbionts shared by all animals analysed displayed high bacterial richness at the phylum level, comparable to that observed in a single sponge hosting about 200 OTUs. This suggests that phylum diversification is fundamental to sponge functioning, and that high redundancy of phylotypes within each phylum likely aids the sponge host in maintaining its repertoire of bacterial phyla in face of local environmental changes or across different developmental stages.

In summary, the recent application of NGS has enabled a better understanding of the “species-level” bacterial diversity in irciniid sponges in comparison with previous molecular technologies. For instance, between 29 and 33 OTUs (99% cut-off) have been reported for *Ircinia* spp. using the cloning-and-sequencing method [34], whereas hundreds to thousands of OTUs have been recovered from irciniids via NGS [14,20,61,62], unmasking the complex microbiomes that inhabit these animals. Nevertheless, previous findings made based on less powerful tools, such as the trends for spatial and temporal stability of these symbiont assemblages, have been confirmed by deep sequencing surveys.

Besides the use of the 16S rRNA gene as a phylogenetic marker to describe sponge-associated bacterial communities, other genes have been selected to uncover putative functions from sponge symbionts, especially those involved in biogeochemical cycling [6]. Among the elements essential to life, nitrogen is one of the most limiting factors affecting ecosystem functioning [63]. The *nifH* gene (which encodes an iron-containing dinitrogenase reductase) has been widely used in diversity assessments of N-fixing bacteria in aquatic and terrestrial environments [64,65,66]. Mohamed and colleagues [21] determined the expression of *nifH* genes in *I. strobilina.* From the cDNA clone library made for this species, 44 *nifH* gene clones were retrieved. They clustered within a group formed exclusively by cyanobacterial phylotypes, indicating that Cyanobacteria symbionts were the prevailing N-fixers in *I. strobilina*. Mohamed and colleagues [24] also disclosed the diversity of aerobic ammonia-oxidizing bacteria (AAOB) associated with *I. strobilina* using cloning-and-sequencing of the *amoA* gene (which encodes the catalytic α-subunit of the ammonia-monooxygenase enzyme). All *amoA* sequences fell into two clusters affiliated with *Nitrosospira* (Betaproteobacteria), suggesting that they were the main nitrifiers in *I. strobilina*. However, no expression of the *amoA* gene could be detected [24]. Hardoim and Costa [62] used the *amoA* gene to unravel the temporal dynamics of ammonia-oxidizing bacteria (AOB) in *S. spinosulus* by PCR-DGGE fingerprinting. Few AOB phylotypes could be detected in the PCR-DGGE profiles of *S. spinosulus* over the three successive years, suggesting that the AOB community in this species was stable over time. As sponges comprise a major portion of the biomass in many coral reefs, microbial mediated nitrogen metabolism within the animal host is expected to have a major impact on the nitrogen budget of these environments [21,24]. More studies are still necessary to ascertain the activity and identity of N-cycling players in marine sponges.

Altogether, Irciniidae species harbour complex and diverse bacteriomes. These are to a large extent maintained by the host species over seasons, years, environmental gradients and geographical regions. Through an in-depth phylogenetic assessment comprising all bacterial 16S rRNA gene sequences (excluding NGS and PCR-DGGE short sequence reads) from Irciniidae sponges publicly available until December 2013, we here substantiate the view of high spatial bacteriome stability in these sponges (Supplementary Figure S1). In fact, bacterial phylogenetic signatures common to the major oceanic regions studied so far could be found within several typical sponge-associated phyla such as the Acidobacteria, Actinobacteria, Bacteroidetes, Chloroflexi, Cyanobacteria and Alpha, Delta and Gamma classes of the Proteobacteria (Supplementary Figure S1a–i). Irciniidae species appear, moreover, to hold their own distinct bacterial communities. These differ from host to host within the same habitat and from the surrounding bacterioplankton communities found in seawater. Thus, intrinsic aspects of the host species—for instance habitat preference, specific geographic distribution and evolutionary history—may cooperatively shape the structure of bacterial symbiont communities in these sponges [34]. Several bacterial taxa associated with Irciniidae species are known for their physiological and metabolic features—of which inhibitory, primary production and nitrogen metabolism capacities can be highlighted—indicating that they play essential roles in the establishment and maintenance of the sponge host.

#### 3.2.2. Bioactivities

Few surveys have described biological activities obtained from bacterial strains—or from their respective metabolite extracts—isolated from Irciniidae species [37,50,67,68]. Although these studies usually do not unveil the identity of the compounds underlying the observed activities, they collectively highlight a broad spectrum of functions of ecological or applied interest within the culturable sponge-associated microbiota. For instance, Thakur and colleagues [68] addressed the hypothetical importance of *I. fusca*-associated bacteria in host defence against bacterial epibionts. In total, 25 isolates were recovered from the sponge surface in five sampling events. Crude sponge extracts were obtained from all events, but only two were active against isolates retrieved from the same sampling. Strains identified as *Micrococcus* sp. and *Bacillus* sp. exhibited antibacterial and antifouling activities, highlighting the potential of both genera to inhibit the growth of epibiotic bacteria on the sponge surface. These results suggest that sponge-associated bacteria can modulate the growth of their own cells as well as their neighbours by producing bioactive metabolites. Esteves and colleagues [37] described the *in vitro* antagonistic activity of 155 distinct genotypes isolated from *S. spinosulus* and *I. variabilis* towards two relevant clinical strains: *Escherichia coli* NCTC 9001 (Gram-negative) and *Staphylococcus aureus* NCTC 6571 (Gram-positive). A few isolates (*n* = 18) were active against both strains, whereas 44 and 27 isolates showed antimicrobial activity towards *E. coli* and *S. aureus*, respectively. The most active genus isolated from Irciniidae species was *Vibrio* with 27 and 12 of the isolates inhibiting *E. coli* and *S. aureus*, respectively. From *S. fasciculatus* collected from the Bay of Bengal (India), five Firmicutes isolates and one Proteobacteria isolate inhibited β-glucosidase activity [69]. Glucosidase inhibitors may be of use in the treatment of viral infections, cancer and genetic disorders [70]. These studies emphasized the biotechnological potential of the culturable bacterial fraction of Irciniidae sponges, encouraging more research into the mechanisms and compounds underpinning such activities.

#### 3.2.3. Bioactive Compounds

Some studies identified the bioactive compounds extracted from Irciniidae-associated bacteria. Several cyclic peptides have been retrieved from *Bacillus* and *Staphylococcus* strains isolated from *I. variabilis* from the Bay of Naples, Italy [71]. Of these, the compound cyclo-(l-prolyl-l-tyrosine) could modulate the activity of the *LuxR*-based quorum sensing system of bacteria [72], whereas all other compounds investigated presented structural similarities to compounds known to interact with *LuxR*-based biosensors [72,73]. Thus, such cyclic peptides may play a role in the marine sponge microbiome as quorum sensing signal molecules [71]. Furthermore, a *Bacillus pumilus* strain was isolated from *Ircinia* sp. and found to produce five surfactin-like substances (cyclic acyldepsipeptides) called bacircines [74,75]. These compounds showed cytotoxic effects on the development of sea urchin eggs. One exhibited antitumour activity against Ehrlich ascites carcinoma and anti-HIV activity [74,75]. These studies demonstrate that the functional screening of compounds extracted from sponge-associated bacteria is a promising, direct route to manifold interesting bioactivities, which nevertheless remains underexplored.

### 3.3. Archaea

In comparison with the multi-layered molecular research undertaken in the past decade on sponge-associated bacteria, fewer surveys have addressed archaeal community composition and diversity in marine sponges. Overall, these studies uncovered less diverse archaeal assemblages from sponges than the corresponding bacterial communities [6,8,76].

#### 3.3.1. Diversity

Phylogenetic inference performed with three distinct archaeal 16S rRNA gene sequences obtained from *Sarcotragus* sp. (Jeju Island, Korea) revealed that they were affiliated to marine group I Crenarchaeota (now known as Thaumarchaeota, see [77,78,79]). Two of them grouped within clusters containing only sponge-derived sequences, but without bootstrap support [80]. Further analysis showed that one of these sequences indeed belonged to a Thaumarchaeota “sponge-specific” cluster (SC175) [76].

Using PCR-DGGE and 454-pyrosequencing, we demonstrated that the archaeal community associated with *S. spinosulus* was stable over three successive years. In 11 out of 12 *S. spinosulus* specimens, one single OTU affiliated to *Nitrosopumilus* dominated the archaeal assemblage, whereas the remaining specimen was dominated by *Cenarchaeum* sp. *Candidatus* Nitrosopumilus maritimus and *Cenarchaeum* sp. are known ammonia oxidizers. This indicates that the replacement of the dominant archaeal symbiont observed for one *S. spinosulus* individual likely does not interfere with the ammonia-oxidation potential in this host [62]. Overall, the diversity of archaea associated with other sponge species was as well encompassed by only few phylotypes [6,76,81,82]. Despite their low diversity, it appears that Archaea, and not Bacteria, are the prevalent players in the process of ammonia-oxidation in marine sponges [83,84,85]. It still needs to be determined which other possible roles, if any, archaeal symbionts play in association with Irciniidae and marine sponges as a whole.

#### 3.3.2. Bioactivities and Bioactive Compounds

Neither *in vitro* bioactivities of archaeal cultures and their metabolite extracts nor identified bioactive compounds from sponge-associated Archaea were found in our literature survey.

### 3.4. Fungi

As pointed out by Webster and Taylor [8], the study of the diversity and functioning of sponge-derived fungi still requires more effort. Fungi have been approached primarily in a cultivation-dependent manner, probably because of their higher amenability to laboratory domestication than that displayed by bacteria and archaea. Whereas this facilitates functional screenings for manifold activities and full genome exploration of the obtained pure cultures, it is felt that comprehensive analyses of fungal diversity and maintenance across temporal and spatial scales could benefit more from dedicated cultivation-independent studies.

#### 3.4.1. Diversity

The diversity of fungi associated with *I. oros* and *I. variabilis* collected at Malta was assessed using several media and incubation for over 30 days at room temperature (~20 °C) [86]. Both sponge species were dominated by *Aspergillus* spp. corresponding to 39.4% and 50%, respectively, of the total isolates followed by *Penicillium* spp. (13.6% and 27%). Whereas 13 and 10 fungal genera were found in *I. oros* and *I. variabilis*, respectively, only five of them were shared by both [86]. The diversity of culturable fungi was also assessed in *Psammocinia* sp. (Sedot-Yam, Israel) [87]. Potato dextrose medium amended with fungicides was used in two approaches: direct plating of inner sponge fragments and direct plating of sponge extracts (“sponge-compressed” method). The Petri dishes were incubated at 25 °C in the dark from three to 30 days. Sequencing of the internal transcribed spacer (ITS) of rRNA genes was used to identify the fungal strains. Overall, 220 pure cultures were isolated and assigned to 85 fungal taxa, of which the majority was obtained using the sponge-compressed method (*n* = 76). Most of the strains (94%) were identified as Ascomycota, whereas Basidiomycota (4%) and Glomeromycota (2%) were minor components of the cultured fungal community. From Ascomycota, 80 “species-level” taxa were recovered and among them 25 were identified as Eurotiales (*Aspergillus* and *Penicillium* spp.), five as Capnodiales (*Cladosporium* spp.), 15 as Pleosporales (*Bionectria*, *Fusarium*, *Phoma*, and *Preusia* spp.) and 35 taxa as Hypocreales (*Acremonium* and *Trichoderma* spp.). When sponge-compressed samples were plated onto medium amended with different fungicides, further 28 taxa that were not obtained with the previous approach could be isolated, thus enabling an increase in the diversity of fungi cultured from the sponge. Using a coverage-based algorithm, the authors estimated that only 15% of the fungi associated with *Psammocinia* sp. could be recovered [87]. Notably, even though both studies used distinct approaches, the fungal diversity obtained from *I. oros*, *I. variabilis* [86] and *Psammocinia* sp. [87] was alike.

#### 3.4.2. Bioactivities

Accompanying their assessment of fungal diversity, Paz and colleagues [87] also investigated the inhibitory activities of fungi isolated from *Psammocinia* sp. using a diffusion bioassay. Thirty-six fungal cultures showed *in vitro* antagonism against at least one of the test fungi (*Alternaria alternata*, *Rhizoctonia solani* and *Neurospora crassafour*) or against the oomycete *Pythium aphanidermatum*. Some isolates (*Trichoderma*, *Acremonium*, *Bionectria*, *Verticillium*, *Penicillium* and *Aspergillus* spp.) were able to secret inhibitory compounds into the growth medium, although those were not identified. It was demonstrated that distinct *Trichoderma* spp. isolated from *Psammocinia* sp. were capable to mycoparasite *Fusarium equiseti* isolated from the same sponge species [87].

#### 3.4.3. Bioactive Compounds

*Penicillium chrysogenum* was isolated from the Mediterranean *I. fasciculata* collected in the Bight of Fetovaia (Italy) and was shown to produce the sorbicillinoid alkaloids sorbicillactones A and B [88]. Sorbicillactone A exhibited remarkable cytotoxic activity towards murine leukaemia lymphoblast (L5178y) cells and anti-HIV activity. This compound is also a promising neuroprotective metabolite. Three other sorbicillinoid compounds (oxosorbicillinol, sorbicillin and bisvertinolone) were identified from *P. chrysogenum*, along with the alkaloids meleagrine and roquefortine C [88]. The isolate *Microascus* sp. K14 from *I. variabilis* also produced the anti-fungal compound fungerin [86].

Irciniids harbour a complex, readily culturable fungal diversity. The potential for uncovering novel fungal-derived bioactive compounds from sponges will become more apparent with the improvement of cultivation and screening techniques and/or the accomplishment of in-depth molecular surveys such as whole genome sequencing of fungal symbionts. This will also improve our understanding of the roles played by sponge-associated fungi within the host.

### 3.5. Other Microeukaryotes

Although diatoms and dinoflagellates have been detected in marine sponges [6], no study involved irciniids*.* There is thus a need to unravel microeukaryote taxonomic, functional and metabolic diversities within sponges as a first step in the pursuit of their biotechnological potential.

In summary, much of the microbial diversity research undertaken so far, considering both marine sponges as a whole and the family Irciniidae, has focused on bacterial communities. Such an interest may reflect the higher relevance of this particular group of symbionts in host fitness. Nevertheless, more effort to unveil the spatial-temporal diversity, composition and abundance of archaeal and microeukaryote assemblages—and their corresponding biological activities—in marine sponges is needed for a balanced picture of the marine sponge holobiont. Within the family Irciniidae, more attention to the genus *Ircinia* was found. Attempts to approach the microbial ecology of *S. spinosulus* have been published only recently [37,38,62], and the highly selective and conserved character of its microbiome makes it a valuable species for comparative studies with other irciniids in the Atlanto-Mediterranean zone and beyond. Besides the recent study by Paz and colleagues on fungal diversity and inhibitory activities [87], very limited information exists on the microbial ecology of *Psammocinia* spp., in spite of their distinct geographical range (Figure 1) and manifold bioactivities (see Supplementary Information). Collectively, approaching the microbiology of these three genera might constitute a rewarding strategy to understanding the microbiology of marine sponges on a global scale with a robust, underlying comparative framework.

## 4. Vertical Transmission of Sponge Symbionts

Vertical transmission is an important mechanism by which sponge-specific symbionts are passed from the parental to the next generation via larvae or gametes. It has been investigated in several sponges and, when present, has usually been interpreted as evidence for an intimate pattern of relationship between host and the transmitted symbionts [30,52,58,89,90,91].

Vertical transmission was proposed as a mode of symbiont inheritance in *I. felix* through the detection of bacteria in adult, larval and juvenile stages of the sponge [30]*.* This study showed, via TEM, that adults contained large and complex bacterial communities, and that high bacterial abundance was also observed extracellularly in the inner region of the larvae, whereas the outer region was almost free of microorganisms. When the host entered the juvenile stage, bacterial cells were found primarily in the mesohyl. Bacterial 16S rRNA gene sequences obtained from *I. felix* adults and offspring (larvae and/or juvenile) belonged to the phyla Acidobacteria, Chloroflexi, Gemmatimonadetes and Proteobacteria (Alpha, Delta, and Gamma classes), and to one lineage of uncertain affiliation. In a subsequent study, Schmitt and colleagues [58] detected several vertically transmitted phylogenetic clusters (VT-clusters), composed by sequences obtained from *I. felix* adults and offspring. VT-clusters affiliated with the Acidobacteria, Chloroflexi, Gemmatimonadetes, Nitrospira and Proteobacteria phyla, suggesting vertical transmission as a mechanism through which a complex bacterial consortium can be formed and maintained within *I. felix*. We still do not know what their function may be in the larvae, nor in settlement, which may occur from a few minutes to several hours after release [92]. Once settled, sponge larvae undergo a rapid metamorphosis to an early juvenile stage. Thus, bacterial symbionts might protect the juvenile from predators by producing antibiotics and deterrent compounds. Arguably, the best example of such an ecological function was obtained by Lopanik and colleagues [93,94,95,96] and Sudek and colleagues [97] while studying the association between the bacterial symbiont *Candidatus* Endobugula sertula and the bryozoan *Bugula neritina*. *Candidatus* E. sertula was found to produce the bioactive polyketides bryostatins. These protected the larvae against predators and were observed in all life stages of *B. neritina*. Bacterial symbionts in sponge larvae may also perform important housekeeping functions or constitute a source of food, since the larvae are unable to take up food particles from seawater [98].

Even though Schmitt and colleagues [58] also described two archaeal VT-clusters from the marine sponges *Agelas conifera* and *Luffariella variabilis*, there is to date no specific documentation of archaeal vertical transmission in Irciniidae sponges, although its existence can be presumed based on available data. Our understanding of fungal acquisition by marine sponges remains otherwise very limited, with neither dedicated studies of this group in sponge larvae or juveniles nor an existing conceptual model on how sponges recruit and maintain these symbionts.

## 5. Bacterial Communication and Signalling Molecules

Quorum sensing (QS) is a mechanism by which microorganisms monitor and regulate their population size through chemical signalling [99]. When quorum-sensing molecules (QSM) released by microorganisms into the extracellular environment reach a threshold concentration, they initiate a signalling transduction cascade that regulates the expression of several target genes in an orchestrated response to the prevailing conditions. QS is a complex mechanism involved in bacterial virulence, swarming motility, conjugal plasmid transfer, biofilm maturation, and antibiotic production and resistance [99]. As such, it affects several activities or areas relevant to human health and the environment (e.g., bacterial multidrug resistance, aquaculture, water purification). These might be impacted by negative feedbacks ensued from QS and its mediation of bacterial metabolism [99,100]. Solutions to QS-derived drawbacks may arise from the activities of the symbiont communities themselves. For instance, from the marine sponge *Luffariella variabilis*, the compounds manoalide, secomanolide and manoalide monoacetate were obtained and showed to be important QS inhibitors [101]. Furthermore, the alkaloids ageliferin and mauritamide B obtained from *Agelas conifera* and *A. nakamurai*, respectively, inhibited the QS of the bacterial reporter *Chromobacterium violaceum* CV017, whereas seven compounds isolated from distinct marine sponges inhibited the QS of *C. violaceum* CV017 and showed antibiotic properties [102].

One of the most studied groups of QSM, *N-acyl* homoserine lactones (AHLs), was investigated in *I. strobilina* inhabiting shallow waters in Key Largo, Florida [22]. Two Alphaproteobacteria and three Gammaproteobacteria isolates were found to produce AHLs, indicating that QS systems play an important role in sponge microbiome dynamics as observed for other systems. The sponge mesohyl constitutes a nutrient rich environment when compared to oligotrophic seawater, thus colonizing bacteria may reach high densities within this microenviroment, allowing them to perform activities mediated by quorum sensing.

Autoinducer-2 (AI-2) is one well-characterized molecule involved in bacterial interspecies communication [99,100]. Briefly, during AI-2 biosynthesis, *S*-adenosylmethionine is transformed into 4,5-dihydroxy-2,3-pentanedione (DPD) by a sequence of three enzymatic reactions, where the last is catalyzed by the enzyme LuxS. Since DPD is unstable, it naturally cyclizes, generating a range of furanones in the presence of water. *Vibrio harveyi*, for example, detects the borate diester form of AI-2 using the LuxP/LuxQ signaling cascade, which may be exclusive to *Vibrio* organisms. AI-2 is associated with a broad range of functions; for instance, virulence in *V. vulnificus* and *V. cholera*, type III secretion, protease production, luminescence, colony morphology and siderophore production in *V. harveyi* [99]. Several surveys detected *Vibrio* spp. associated with different marine sponges [37,49,50,51,103,104,105,106], indicating that they may be capable of performing an array of functions within the sponge host. From *I. strobilina* (Key Largo, USA), 10 out of 40 isolates were identified as *Vibrio* spp. based on 16S rRNA gene sequencing [25] and found to be closely related to *V. harveyi* (*n* = 5) and *V. campbelli* (*n* = 4). Notably, all 10 *Vibrio* isolates were capable of synthesizing AI-2 molecules. Furthermore, 30 distinct *Vibrio luxS* gene sequences were identified, of which 28 were closely related to the *luxS* gene of *V. harveyi* and the other two were affiliated with the *luxS* gene of *V. parahaemolyticus*. Interestingly, two *luxS* gene sequences were distantly related to any known *Vibrio luxS* sequence. These were proposed to represent a sponge-specific *luxS* cluster [25].

**Figure 6:**
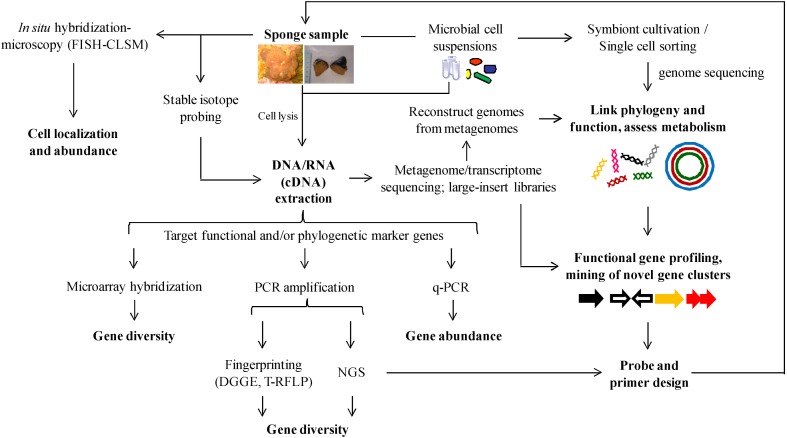
Integrated molecular approach for the study of symbiont communities in Irciniidae sponges.

Two of the five known classes of QSM have already been detected in the bacterial community associated with *I. strobilina*, and more may be found as research advances. Considering the complexity of the Irciniidae microbiome, future investigations involving, for example, full genome sequencing of symbionts and deep sponge metagenomic mining (Figure 6) for QS regulatory genes may result in the discovery of other classes of QSM. It is too early to determine the precise role(s) of QS in gene regulation and metabolism of both symbionts and host. The application potential of QS regulation can be nevertheless exemplified from other host-microbe associations in the marine realm. For instance, *Shewanella* sp. strain MIB010, isolated from the intestine of the ayu fish, is an effective agent against the QS-regulated biofilm formed by the fish pathogen *V. anguillarum* [100].

## 6. Diseases Affecting Irciniids

Many studies have shown that microorganisms might also cause disease in marine sponges [107]. In 1884, a disease affected Indian Ocean *Ircinia* spp., whereby fungal filaments were shown to destroy the sponge body, leaving only the hard spongin fibres [107]. In addition, the abundance of *I. variabilis* in the benthic community of the Marsala Lagoon (Italy) decreased from 6.9% in 1986 to 3.0% in 1989, due to a disease characterized by white patches on the surface of affected individuals [108]. Several *Ircinia* specimens from Guigalatupo, Panamá, were lost over five censuses performed from 1984 to 1998, whereby spreading lesions were seen in *I*. *felix*, *I*. *campana* and *I. strobilina* [109]. A mass mortality event was reported in 1994 affecting *S. spinosulus* and *Ircinia* sp. in the Mediterranean Sea, characterized by the decay of their spongin filaments [107]. Four more recent disease outbreaks affected Irciniidae species in the Mediterranean, Ionian and Adriatic Seas [59,110,111,112], eventually with severe consequences for the infected populations [111]. Whereas loss of photosynthetic cyanobacteria was documented among diseased specimens in one case [111], higher abundance of *Vibrio* spp. was observed in another [59]. In one study [110], a twisted rod bacterium was proposed as the etiological agent because it penetrated the sponge body, proliferated, and outcompeted a large range of bacterial symbionts. Shifts in symbiont communities towards a more virulent state because of elevated seawater temperature have been hypothesized to trigger the mass mortality events [59,110,111,112]. In this context, Pita and colleagues [113] subjected *I. fasciculata* and *I. oros* to aquaria experiments under high temperature (25 °C), food shortage (0.1 μm-filtered seawater) and both factors in combination. T-RFLP analysis showed that the sponge-associated bacterial communities did not change after acclimation time and during the next three weeks of experiment. TEM revealed that the ectosome of *I. fasciculata* was dominated by *Candidatus* S. spongiarum. Under high temperature and food shortage, both healthy and damaged cyanobacterial cells were observed. For *I. oros* under all treatments, no signs of sponge or bacterial cell degradation or cyanobacterial cells were detected. Thus, high temperature and food shortage up to three weeks did not disrupt the bacterial communities associated with both sponge hosts. The authors suggested that these factors could not alone explain the mass mortality events registered for *Ircinia* spp. in the Mediterranean Sea [113].

To date, few surveys have identified bacterial species as the etiological agents of disease in sponges [59,107]. More often, a clear cause-effect relationship between agent and disease is difficult to achieve [107,114]. More research is needed to improve our capability of identifying etiological agents and unravelling how biotic and abiotic features influence marine sponge disease incidence in a changing climate.

## 7. Experimental Microbial Ecology

Sponge microbiology is a fertile field for experimentation. Most of the first, experimental sponge microbial ecology studies kept the host animals for varying periods in aquaria, and tested whether the structure and function of their associated bacterial communities shifted during the maintenance periods [23,115,116]. Surveys using other sponge hosts reported changes in bacterial community composition of specimens kept in aquaria over six months and two years [115,116]. In contrast, evidence exists of recovery within Irciniidae symbiont communities back to the *in-situ* stage after some time in captivity [23]. Specifically, Mohamed and colleagues applied molecular techniques to monitor bacterial communities associated with *I. strobilina* maintained in aquaculture [23]. Thirty-five OTUs were retrieved from wild specimens, while 48 and 47 OTUs were recovered from specimens kept in aquaria for three or nine months, respectively. Overall, these OTUs were affiliated to five or six bacterial phyla depending on the category (wild, 3- and 9-month sponges). Although the highest diversity was observed for the 3-month sponges, 9-month sponges showed an intermediate diversity between wild and 3-month sponges, which suggested acclimatization of the holobiont to the aquaculture system. From the sponges maintained in aquaculture for three and nine months, chemical fingerprints of small molecules including primary and secondary metabolites extracted with ethanol were analysed by liquid chromatography-mass spectrometry and compared to the wild type. The analyses revealed no major changes in the natural product profile of *I. strobilina* upon transfer to aquaculture [23]. This suggests that, although the bacterial community from aquarium-maintained *I. strobilina* differed from those of the wild specimens along the assay, the major functions were retained. This could be explained by the functional redundancy of sponge bacterial symbionts [117,118]. So far, no study approached sponge-associated archaeal or fungal communities during maintenance in aquaria. 

It is still to be determined whether sponge microbial communities can be successfully stabilized in aquaculture, and longer surveys may shed more light. Technical problems in maintaining sponges in captivity have hindered experimental progress, which is fundamental to elucidate how the marine sponge microbiome might be affected by e.g., temperature increase [119,120], water acidification [121] or invasive pathogens (see Section 6 above). However, *Ircinia* spp. have shown amenability to experimental handling [23,113], a feature that reinforces their suitability as a model taxon in microbiology and biochemistry studies.

## 8. Metagenomics-Based Discovery of Secondary Metabolites Biosynthetic Gene Clusters

Polyketides are usually found in sponges and comprise a structurally varied class of compounds that have attracted considerable attention due to their highly potent cytotoxicity [122]. Polyketide synthases (PKS) are multifunctional enzymes of typical microbial origin that catalyse polyketides biosynthesis. However, the complexity of the bacterial assemblages associated with marine sponges makes identification of the actual PKS producers difficult. Some techniques have been applied to overcome these limitations; for instance, cloning-and-sequencing, genome, single-cell genome and metagenome mining of PKS encoding operons (Figure 6). Targeting the biosynthetic gene clusters, two pederin-type PKS systems putatively involved in the synthesis of antitumour polyketides were located in the metagenomic DNA of *Theonella swinhoei* [123]. Likewise, metagenomic libraries generated from cell fractions enriched for filamentous bacteria associated with *Discodermia dissoluta* revealed that they consisted of several non-ribosomal synthase (NRPS) as well as mixed PKS-NRPS gene clusters [124]. Moreover, genomic mining of two bacterial cells sorted from *Aplysina aerophoba* and belonging to the phyla Poribacteria and Chloroflexi revealed Sup-PKS (“sponge symbionts ubiquitous PKS gene”) and NRPS gene clusters in the screened genomes, respectively [15]. Single-cell genomics further uncovered at least two PKSs, one of which affiliated with the sponge-specific “Sup-type PKS” from a Poribacteria cell sourced from *A. aerophoba* [125]. Collectively, these studies demonstrate the wide applicability of metagenomics to studying the function and bioactive potential of marine sponge symbionts.

Within Irciniidae sponges, psymberin was isolated from *Psammocinia* aff. *bulbosa* collected at Milne Bay (Papua New Guinea) [126]. The complete sequencing of three fosmids revealed common features of bacterial genomic architecture: no introns were found, the genes were preceded by Shine-Dalgarno sequences, the space between genes suggesting that the transcribed mRNA was polycistronic and the close relationship to genes exclusively from bacteria. Nevertheless, the low similarity of these genetic signatures to genes from *Pseudomonas* sp. or other prokaryotes hampered the correct identification of the bacterial producer. Finally, a strong correlation among bacterial abundance, the presence of Poribacteria and Sup-PKS was observed in the Caribbean *I. felix*, and it was proposed that Poribacteria was most likely the producer of mid-chain-branched fatty acids (MBFAs) from this sponge [127].

## 9. Cultivation of Irciniidae Species

Even though marine sponges produce the largest variety of secondary metabolites in marine ecosystems, few compounds have reached commercial production. This is because of the normally minute amounts readily found in the sponge body and of the impossibility to collect sufficiently large quantities of sponge biomass from their natural habitats, which is intolerable given the foreseen impacts on the marine environment and natural sponge populations. To circumvent this, many techniques have been developed to cultivate sponge species [6,128,129,130]. Below we highlight attempts to sponge cultivation using Irciniidae. We divide these attempts into three modalities: sponge mariculture, cell culture and larviculture.

### 9.1. Sponge Mariculture

In a preliminary study, Wilkinson and Vacelet [131] failed to transplant *I. variabilis* collected from Endoume (near Marseille) at 3–4 m from well-shaded cave walls and rock cliffs to a 7 m deep rock shelf around 30 m from the sampling place. All specimens died a few days after sampling, most likely due to severe damage caused by sampling, cutting and sewing of *I. variabilis* into plastic plaques.

Duckworth and colleagues [132] examined the feasibility of cultivating *Psammocinia hawere in situ*. Fifty specimens of *P. hawere* were collected from 10–20 m at “Ti Point Reef” (New Zealand). They were cut into cubes of four different sizes and transplanted to two locations, along with small whole sponges as undamaged controls. Three depths (5, 10 and 17 m) and two culture systems were used, and the experiment was conducted in summer (82 days) and winter (88 days). The survivorship was considered high (276 out of 360 explants), even though 2/3 of the explants (226) lost weight. *P. hawere* explants transported to deeper (10 and 17 m) waters in winter showed the highest growth and survivorship rates. This was related to the lower UV radiation as well as the cooler water temperatures, which helped to accelerate pinacoderm healing. *P. hawere* is known to incorporate detritus from the sediment into its body or fibres, which may have enhanced the surface consolidation during the healing process. Growth and survivorship improved with the size of the explant and the proportion of the intact pinacoderm. Independently of the culture systems used, *P. hawere* displayed better growth and survivorship at 10 and 17 m than at 5 m.

Van Treeck and colleagues [133] sampled 52 and 100 specimens of *I. variabilis* at the north-western coast of Corsica in summer and spring, respectively. They were transplanted to naturally formed sand patches in-between seagrass meadows at 15 m. Prior to transplanting, the sponge specimens were cut into different sizes underwater. The explants were placed between two frames, closed, mounted and transported to the experimental sites. The survival rates of *I. variabilis* were high within the first six months, followed by a reduction of 25% in population size, most likely due to a strong storm, after which the remaining sponge continued to grow. The specimens stocked in spring survived better. In the first 12 months, *I. variabilis* biomass increased by 90%; however, there was great variation between maximum and minimum rates (220 and −43%, respectively) [133].

In another study [134], *I. ramosa* was collected from Bone Lola reef (Indonesia) and cut *in situ* into explant sizes of around 30 mm where at least one side contained exopinacoderm. A polyethylene rope was passed through the sponge body, each rope carrying around nine explants. Ropes were attached to PVC frames that were placed horizontally ~20 cm above the reef bottom between 12–15 m deep, and secured with iron pegs at the exposed side of the submerged reef. A high survival rate of 92% was observed for *I. ramosa* during the four months. A slight but significant increase in length was detected, and a change in shape was observed which could not be precisely measured. However, only few explants grew during the trials [134].

### 9.2. Sponge Cell Culture

In a series of studies, de Rosa and colleagues [135,136,137] meticulously monitored the chemical composition of *I. muscarum* cells grown on a simple culture medium. Cell suspensions from *I. muscarum* (Gulf of Naples, Italy) were resuspended in sterile seawater complemented with antibiotics during the first two weeks of cultivation. The cultures were incubated at 18 °C in the dark and at 22 °C in the light and every third day the medium was renewed. From the third week onward, cultures were kept in Dulbecco’s modified Eagle’s medium supplemented with glucose. This medium was again renewed every third day. One week after inoculation, cells were observed attached to the bottom of culture chambers along with some aggregates. Cells were actively dividing in both culture conditions and stationary phase was reached after four days. Overall, 17 sterols were recovered*.* Around 90% of the total free sterols in cultured *I. muscarum* were represented by Δ^5−7^ sterols; from which 7-dehydrocholesterol, ergosterol and 7-dehydrositosterol prevailed. The medium was then supplemented with water-soluble cholesterol, which increased the number of observed cells by 70%. Oleic acid was one of the main acids in the lipids of the intact sponge and was found in low concentration in cultured cells. When the media were supplemented with oleic and linoleic water-soluble fatty acids, no growth promotion was observed. However, they were completely metabolized by the cells. The concentration of total lipids was higher in the intact animal, and cells developed in the dark had higher total lipids than those grown under light. Several structurally distinct groups of volatile compounds were observed, and their concentrations indicated that bacteria (from diet or symbionts) could be involved in their formation. A number of free amino acids were detected in the intact sponge, whereas practically none was found in the cell cultures. The major difference in carbohydrate composition between intact and cultured sponge cells was the presence of γ-lactones of 3-desoxy-arabino-hexonic acid and 3-desoxy-ribohexonic acids in higher concentrations in the cell cultures, suggesting shifts in their carbohydrate metabolism. The cultured cells did not produce any secondary metabolites as the major cell energy supplies had likely been allocated to the biosynthesis of primary compounds [135,136,137].

### 9.3. Sponge Larviculture

With the aim of culturing sponges from larvae, de Caralt and colleagues [138] transported mature individuals of *I. oros* sampled from I’Escala (Mediterranean Sea) to an open aquaculture system, where they released larvae after about 45 days. Swimming larvae were then collected and transferred to six-multi-well dishes. These were placed in aquaria with filtered seawater (0.7 µm pore diameter) at the same temperature of the field (20 °C). Settlement of the larvae on the six-multi-well dish bottom started after 24 h and larvae metamorphosed into juveniles after five to seven days. An exhalant tube should be formed after this period; however, no visible inhalant/exhalant orifices were observed among the settlers of *I. oros* and, at this point, most juveniles died before the skeletal fibres could be formed. Although settlement success was considered high (94.5%), no survival of *I. oros* specimens was observed after 50 days of experiment. Success in settlement could be related to favourable conditions such as still water and no substrate competition found by the larvae in the laboratory [138]. The first two weeks of culture showed the maximum increase in area, but this was primarily due to rearrangement of the biomass during metamorphosis, rather than true growth [138]. Therefore, unknown and specific environmental cues are likely needed to enable full development of sponge larvae into mature individuals in the laboratory.

From the three modalities approached above, for Irciniidae species mariculture is apparently the most feasible (host) culturing methodology, rather than cell or larvae cultivation. However, whether irciniids will produce secondary metabolites under these conditions remains to be tested. Osinga and colleagues [128] reported on five sponge species that, grown in mariculture, were able to keep the biosynthesis of target metabolites, demonstrating that this approach can be rewarding. Optimization of explant size, depth of transplantation, temperature ranges, and exposure to light and water currents might improve sponge productivity and bioactive compound availability. Besides mariculture, *ex-situ* culture (*i.e.*, aquaculture) of adult specimens and semi-synthesis of metabolites are promising alternatives. In the former, the sponge grows under controlled conditions in flow-through or recirculation systems, and the experimental costs and yields of bioactive compounds would be comparable to mariculture. In the latter, a biosynthetic precursor produced by a genetically modified bacterium triggers a limited number of further chemical reactions to the synthesis of the final product. For instance, using the antibiotic cyanosafracin B, which was obtained from *Pseudomonas fluorescens* via bacterial fermentation, an analogue of the antitumor compound ecteinascidin 743 isolated from the tunicate *Ecteinascidia turbinata* was effectively synthesized [130]. Combining biological and chemical approaches might be an effective way to cultivate sponges and recover their secondary metabolites.

## 10. Concluding Remarks and Outlook 

Irciniidae-microbe interactions are pivotal to host health and have been evolving since the hosts’ origins. In recent years, a few fundamental issues regarding these interactions have been successfully addressed. For instance, we now know that distinct bacterial symbionts are selectively enriched within the sponge body and, eventually, vertically transmitted, and that such symbiont communities are stable along time and across geographical regions. Nevertheless, many other questions remain unanswered. How full symbiont genomes evolve across space and time, both within individuals of the same species and between different host species? Do single gene-based analyses (e.g., 16S rRNA gene sequencing) suffice to completely determine microbial diversification and evolution in marine sponges? What are the functions played by the symbionts in host health and functioning, and which symbiont does what? How do the vertically transmitted microorganisms affect larval settlement and juvenile establishment? What are the benefits of quorum sensing for the host? Are secondary metabolites produced by symbionts effectively involved in host’s chemical defence? Is there a true etiological agent (or many) underpinning disease outbreaks in Irciniidae species? Is the existent technology for their laboratory maintenance robust and widely applicable? Furthermore, the majority of microbiology studies of Irciniidae species have so far mainly determined bacterial community structures in these hosts. Although useful in shedding new light on our understanding of the diversity and stability of bacterial assemblages in marine sponges, these inventories usually do not unravel the biotechnological potential of the bacterial partners. Other microbial associates, such as archaea, fungi and other microeukaryotes—and their biotechnological value—have been left almost unaddressed. Thus, only by integrating the full holobiome in future surveys will a more comprehensive picture emerge, allowing better understanding of host function. Currently, several advanced molecular approaches such as metatranscriptomics, metaproteogenomics and single-cell genomics, may help answer these questions (Figure 6). Indeed, their recent application has already been useful in disentangling the phylogeny-function dilemma, and in unveiling the life-style and secondary metabolite biosynthesis capacity of several, so-far uncultured, marine sponge symbionts [15,125,139,140,141]. Finally, few alternative culturing protocols have been attempted so far for marine sponge microbial associates. The spectrum of microorganisms cultured from these hosts is likely to grow in the next few years as researchers develop dedicated protocols to the capture of “hard-to-culture” symbionts. In this regard, ever-increasing genetic datasets retrieved via cultivation-independent methods can help in the identification of symbiont metabolic pathways and physiological demands that could be explored in future cultivation trials. Given the aforementioned challenges, a joint interdisciplinary effort is clearly needed, from fields ranging from microbiology to chemistry, and from genetics to zoology, to advance the knowledge of the oldest extant metazoan-microbe interaction system on Earth. Ideally, this would couple laboratory experimentation with integrative systems biology strategies; therefore, we reinforce the need to select marine sponge models, and provide a multitude of reasons in favour of the Irciniidae family as one such model.

## Supplementary Files

Supplementary File 1

Supplementary File 2
