# Supplementary material for: Microbial Communities and Bioactive Compounds in Marine Sponges of the Family Irciniidae—A Review
Source: Mar Drugs. 2014 Sep 30;12(10):5089–122. doi: 10.3390/md12105089 (PMC4210886; doi:10.3390/md12105089)
Supplement: Supplementary File 1 [file marinedrugs-12-05089-s001.pdf]

Supplementary Information

Secondary Metabolites Retrieved from Irciniidae Specimens and the Possible Role of Associated Microorganisms in Their Biosynthesis

Increasing research interest in the sponge-associated microbiota can be to a large extent attributed to the production of an enormous diversity of biologically active secondary metabolites by marine sponges, especially in the class Demospongiae [1]. Some studies suggested that certain bioactive compounds retrieved from marine sponges; for instance, complex polypeptides and nonribosomal peptides, are likely to be synthesized by the symbiont bacteria. This is due to their high resemblance with metabolites known to be produced by bacteria, or to the fact that they belong to a class that is commonly found in bacteria [2–6]. Notably, species within the family Irciniidae host several metabolites primarily belonging to the large terpenoid and polyketide classes of natural products. Many of these metabolites have been directly extracted from the host animal and shown interesting biological activities. They are described below, along with comments on their potential microbial origin whenever possible. It is not the scope of this review to provide the molecular structure of the compounds described below. These structures can be accessed in the original reports covered by our survey.

1. Terpenoids and Prenylated Compounds

Terpenoids are a class of compounds formally derived from isoprene units and classified based on the number of carbon atoms in their molecular structure—e.g., monoterpenoids (C_{10}), sesquiterpenoids (C_{15}), *etc* [7,8]. There are over 24,000 known terpenoids and many have been applied in food and pharmaceutical industries [8,9]. The antimalarial Artemisinin derived from the plant *Artemisia annua* and the anticancer paclitaxel (Taxol®) synthesized by the endophytic fungus *Penicillium raistrickii* in the bark of *Taxus brevifolia*, which is being used to treat ovarian, lung and breast cancers, and head and neck carcinoma and melanoma [8], are examples of terpenoids used in human health research. Furthermore, paclitaxel is in clinical trials for other forms of cancer [10]. In 2002, the worldwide sales of terpenoids-based pharmaceuticals were around 12 billion US dollars [8]. Besides, terpenoids are also part of other compounds like meroterpenoids, which encompass terpenoid- and non-terpenoid derived fragments such as chromanols, polyprenylated quinones and hydroquinones possessing a wide range of biological activities [11]. Terpenoids dominate the chemistry of marine cnidarians, while terpenoids, quinones and hydroquinones have been detected in marine sponges and in marine bacteria [11–18]. In the following, we describe terpenoids and prenylated quinones/hydroquinones isolated from Irciniidae species as well as their cytotoxic activities.

1.1. Anti-Viral Activity

The compounds penta-, hexa- and heptaprenylhydroquinone 4-sulphates were obtained from *Ircinia* sp. (Norfolk Ridge region, New Caledonia) and showed to inhibit HIV-integrase enzymes [19]. Although showing promising results as anti-HIV drugs, heptaprenylhydroquinone and its methylated derivative were found to be toxic to mammalian cells in culture [20]. A MeOH/toluene extract from

Sarcotragus sp. showed significant *in vitro* antiviral activity towards *Herpes simplex* I and *Polio* type I [21].

1.2. Antimicrobial Activity

The mixture of variabilin and strobilinin isolated from *I. strobilina* showed high activity against *Staphylococcus aureus* and *Bacillus subtilis* [22,23]. Palominin isolated from *Ircinia* sp. (Palomino Island, Puerto Rico) inhibited the growth of *Proteus vulgaris* and *Shigella flexineri* [24]. The 2-octaprenyl-1,4-hydroquinone isolated from *I. spinosula* (note by authors: non-valid taxon. Accepted name: *Sarcotragus spinosulus*) (Saronicos Gulf, Greece) was the most active metabolite against the development of marine bacteria and fungi compared to compounds obtained from *I. oros* and *I. variabilis* [25]. Sulfircin obtained from a deep-sea *Ircinia* sp. (Andros, Bahamas) was active against *Candida albicans* [26].

1.3. Anti-Crustacean Activity

Some terpenoids were highly toxic in the *Artemia salina* shrimp test. These encompassed palominin recovered from *Ircinia* sp. (Palomino Island, Puerto Rico; [24]), ircinin-1 and -2 (furanosesterterpenes) isolated from *I. oros* (North Adriatic Sea; [27]), and palinurin and fasciculatin sulphates recovered from *I. variabilis* and *I. fasciculata* (Currently accepted taxon: *Sarcotragus fasciculatus*. For the sake of clarity, in this review we will keep the source name *Ircinia fasciculata* whenever referring to previous studies.) (Bay of Policastro), respectively [28]. From *Sarcotragus* sp., sarcotin P (norsesterterpenoids) was also effective in the *A. salina* shrimp test [29]. Additionally, three sulphated 2-prenylhydroquinone derivatives retrieved from *I. spinosula* (note by authors: non-valid taxon. Accepted name: *Sarcotragus spinosulus*) (Sutomixica, Zadar, Croatia) exhibited greater activity in the *A. salina* shrimp assay than the corresponding hydroquinones. This indicates that these compounds play a defensive role against macro-symbionts, which were indeed absent in the host sponge [30].

Ircinin-1 and -2 (furanosesterterpenes), obtained from the Mediterranean *I. oros*, were very active in a settlement inhibition assay of the cyprids *Balanus amphitrite*, whereas the acetates of the compounds 2-octaprenyl-1,4-hydroquinone and 2-(24-hydroxy)-octaprenyl-1,4-hydroquinone isolated from *S. spinosulus* (Saronicos Gulf, Greece) reduced the settlement of *B. amphitrite* larvae [25,31,32].

1.4. Activities against Vertebrates (Fish and Sea Urchin)

Tsoukatou and colleagues [25] demonstrated that the mixture of ircinin-1 and -2 obtained from *I. oros* was responsible for the anti-feeding behaviour of the generalist predator fish *Thalassoma pavo*. Palinurin has been recovered from *I. variabilis* at different sampling sites [28,33,34] and from *Psammocinia* sp. (Korea; [35]), and moderate cytotoxicity was observed in the fish lethality test [28]. Reef fishes avoided the crude extract of *I. strobilina* incorporated into carrageenan or calcium alginate strips at its natural concentration. Further analysis revealed variabilin as the deterrent agent, whereas pure variabilin added to calcium alginate was responsible to deter the feeding of natural assemblages of reef fishes [36,37]. Variabilin also inhibited the feeding of the generalist predator fish *T. pavo* [25]. Fasciculatin isolated from *I. fasciculata*, *I. oros* and *I. variabilis* sampled at different

locations [28,31,38,39] showed high toxicity in the fish lethality assay [28]. Moreover, the furanosesterterpene tetronic acid (FTA) suvanine recovered from *Ircinia* sp. (Fiji Island) displayed toxicity to goldfish and inhibited cell division of the sea urchin [40]. Freeman and Gleason found FTAs in the inner body of *I. felix*, whereas in *I. campana* there was no significant difference between the concentration of FTAs in the inner and outer layers [41]. This observation contradicts the previous idea that the antipredator compounds should be in the outermost 2 mm of the sponge body. The authors suggested that this high concentration of FTAs in the interior of the sponge may act to protect this important region where physiological processes take place. In another survey, Freeman and Gleason [42] demonstrated that extracts from inner and outer compartments of *I. campana* did not differ in the capacity to deter generalist temperate reef fishes and the widespread sea urchin, whereas extracts from the inner region of *I. felix* did not show enhanced deterrence to generalist reef fish and one species of sea urchin. These results indicate that an increase in the concentrations of the defensive chemical did not improve the protection of the sponges from predators, and that other factors might play a role in the defensive compound allocation.

1.5. Anti-Cancer Activity

Several compounds recovered from *Sarcotragus* sp. showed marginal to significant cytotoxicity towards a panel of five human cancer cell lines (A549 (colon), SK-OV-3 (ovarian), SK-MEL-2 (skin), XF498 (Central Nervous System - CNS) and HCT-15 (colon)). These comprehended sarcotins A to J, M, N and O; sarcotins K and L (bisfuranoditerpenes) obtained as an inseparable mixture; ent-kurospongin; sarcotragins A and B (trinosesterterpene lactans); epi-sarcotins A, B and F (pyrrolosesterterpenoids); epi-sarcotrine A and B; ircinin-1 and -2; (7*E*,12*E*,18*R*,20*Z*)-variabilin; (8*E*,13*E*,18*R*,20*Z*)-strobilin; sarcotrine A to F; *epi*-sarcotrines A to C (pyrrolosesterterpenoids); and iso-sarcotrines E and F (pyrrolosesterterpenoids) [29,43–48]. Ircinin-1 isolated from *Sarcotragus* sp. inhibited the proliferation of the skin cancer cell line SK-MEL-2 [49]. Further, the crude extract containing sarcotragins A and B displayed moderate cytotoxicity towards the leukaemia cancer cell line K652 [48]. Moreover, palinurin and isopalinurin were recovered from a Korean *Psammocinia* sp. The former exhibited moderate selective cytotoxicity against the SK-MEL-2, A549, SK-OV-3, XF498 and HCT15 cancer cell lines [35]. Fasciculatin obtained from *I. fasciculata* (Bay of Naples, Italy) inhibited the growth of the human cancer cell lines MCF-7 (breast), NCI-H640 (lung) and SF-268 (CNS) on a dose-dependent manner [39].

The kohamaic acids A and B (bicyclic sesterterpenoids) isolated from *Ircinia* sp. exhibited cytotoxicity against the P388 leukaemia cell line [50]. From a Taiwan *Ircinia* spp., Su and colleagues [51] isolated 15-acetylirciformonin B and 10-acetylirciformonin F, which exhibited strong and moderately cytotoxic activity, respectively, against the K562 (chronic myelogenous leukaemia), DLD-1 (colon adenocarcinoma), HepG2 and Hep3B (liver carcinoma) cancer cell lines. The authors suggested that the furan moiety was essential for the cytotoxic activity of C_{22} furanoterpenoids. Irciformonin B and F were also retrieved and the former showed significant cytotoxicity against the K562, DLD-1 and HepG2 cancer cell lines, whereas the latter was active against the HepG2 cancer cell line [51]. Further, heptaprenyl- and octaprenylhydroquinones isolated from *S. spinosulus* (Callejones, Ceuta) inhibited cell metabolism and the number of cancer cells (K562) by, most likely,

inducing apoptosis [52]. Irciformonin C and D (trinorsesterterpenoids) isolated from *I. formosana* (Taiwan), exhibited mild cytotoxicity against WiDr (colon adenocarcinoma) cancer cells [53].

Wätjen and colleagues [54] retrieved hexa- and nonaprenylhydroquinone from *S. muscarum* and heptaprenylhydroquinone from *I. fasciculata* collected in Mersin and Fethiye (Turkey), respectively. These compounds inhibited nuclear factor-kappa B (NF-κB)-signalling in H4IIE hepatoma cells, and heptaprenylhydroquinone was the most active. Hexa- and heptaprenylhydroquinone disrupted the extracellular-signal regulated kinase signalling pathway by inhibition of the epidermal growth factor receptor (EGF-R), and heptaprenylhydroquinone also inhibited the activity of other kinases. Thus, the heptaprenylhydroquinone might be especially useful for the development of anti-cancer drugs.

From a Korean *Psammocinia* sp., three novel scalarane sesterterpenes were obtained—12-deacetoxy-23-hydroxyscalaradial (i), 12-dehydroxy-23-hydroxyhyrtiolide (ii) and 12-*O*-acetyl-16-deacetoxy-23-acetoxyscalarafuran (iii) along with the known compounds 12-deacetoxy-23-hydroxyheteronemin (iv); 12-deacetoxy-23-acetoxy-19-*O*-acetylscalarin (v); 12-deacetoxy-23-*O*-acetoxyheteronemin (vi) and 12-deacetoxyscalaradial (vii) [55]. Among them, compound (iv) showed the most potent activities towards human kidney carcinoma (ACHN), epithelioid carcinoma (PANC-1) and human pancreatic carcinoma cell lines, and its activity was significantly stronger than the general anticancer drugs used as positive controls. Compounds (v) and (vi) were also more cytotoxic than the control drugs employed [55].

1.6. Anti-Inflammatory Activity

The search for selective inhibitors of phospholipase A_2 and their capacity to control inflammatory processes appeared as an alternative to non-steroidal anti-inflammatory drugs. From *S. spinosulus* (Bay of Naples, Italy), the compounds 2-octaprenyl-1,4-hydroquinone and 2-(24-hydroxy)-octaprenyl-1,4-hydroquinone were recovered, and the greatest inhibitory effect occurred on a human recombinant synovial enzyme in a concentration-dependent manner compared to snake and bee venom [56]. Moreover, octa- and nonaprenylhydroquinone sulphates retrieved from *Sarcotragus* sp. (Cleveland Bay, Australia) inhibited α1, 3-fucosyltransferase VII and thus may be strong drug candidates to treat inflammatory diseases [57]. Instead, the compound Sch599473 isolated from *Ircinia* sp. exhibited weak inhibitory activity in the CCR7 receptor binding assay which is involved in a biological cascade including inflammatory, allergic and metastasis processes [58]. From *I. spinosula* (note by authors: non-valid taxon. Accepted name: *Sarcotragus spinosulus*) collected at Saronicos Gulf (Greece) the compounds 2′-(28-hydroxy)-heptaprenyl-1′,4′-hydroquinone, 2-hexaprenyl- and 2-heptaprenylhydroquinones were recovered and displayed anti-inflammatory activities [59].

1.7. Inhibitors of Protein Kinases

From a deep-sea *Ircinia* sp. (Norfolk Ridge region, New Caledonia) the compounds penta-, hexa- and heptaprenylhydroquinone 4-sulphates were recovered and inhibited the tyrosine protein kinase [19]. The cheilanthanes sesterterpenoids 25-hydroxy-13(24),15,17-cheilanthatrien-19,25-olide; 13,16-epoxy-25-hydroxy-17-cheilanthen-19,25-olide; 16,25-dihydroxy-13(24),17-cheilanthadien-19,25-olide and 25-hydroxy-13(24),17-cheilanthadien-16,19-olide, retrieved from an Australian

Ircinia sp., inhibited the serine protein kinases MSK1 (mitogen and stress activated kinase) and MAPKAPK-2 (mitogen activated protein kinase) which are involved in signal transduction [60].

1.8. Other Activities

Sarcochromenol sulphate A and sarcohydroquinone sulphates A to C isolated from *S. spinosulus* (Tasmanian Sea) repressed the activity of Na^+, K^+ -ATPase from the rat brain [61].

The bicyclic sesterterpenoid kohamaic acid A, retrieved from *Ircinia* sp., was a potent DNA polymerase inhibitor [50]. Additionally, 11 analogues of kohamaic acid A have been synthesized in the laboratory and it was proposed that the carboxylic acid was important for the inhibition of DNA polymerase [62].

From *S. spinosulus* (Saronicos Gulf, Greece) the compounds 2-octaprenyl-1,4-hydroquinone and 2-(24-hydroxy)-octaprenyl-1,4-hydroquinone were isolated [25,31]. The former showed strong interaction with 1,1-diphenyl-2-picrylhydrazyl radical (DPPH) and had a moderate effect on lipid peroxidation, whereas the latter interacted extensively with DPPH and exhibited a significant effect against lipid peroxidation [25]. Methanolic extracts from *I. fasciculata* collected at Kremer (Turkey) significantly scavenged nitric oxide (NO) and superoxide (SO) radicals [63]. These three free radicals (DPPH, NO and SO) are involved in the pathogenesis of diabetes, arteriosclerosis, cardiovascular diseases, cancer and several neurodegenerative disorders [63].

Irciformonin I (trinorsesterterpenoids) isolated from a Taiwan *I. formosana* showed significant inhibition on peripheral blood mononuclear cell proliferation induced by phytohemagglutinin [64].

The compounds chromarols A to D isolated from a Papua New Guinea *Psammocinia* sp. were shown to be potent and effective inhibitors of human 15-lipoxygenase which is responsible for the signal pathway of atherosclerosis [65].

From an Australian *Ircinia* sp. and *Psammocinia* sp., three compounds (glycinyl lactam sesterterpene/ sesterterpene tetronic acids) were retrieved, from which (12*E*,20*Z*,18*S*)-8-hydroxyvariabilin may lead the development of new drugs to treat temporal lobe epilepsy, while 8-hydroxyircinialactam A and B could be helpful in the treatment of movement disorders [66]. The compounds (−)-ircinianin, (−)-ircinianin sulphate, (−)-ircinianin lactam A, (−)-ircinianin lactam A sulphate, (−)-oxoircinianin, (−)-oxoircinianin lactam A, and (−)-ircinianin lactone A were obtained from three Australian *Psammocinia* sp. The glycinyl lactams (−)-ircinianin lactam A and (−)-oxoircinianin lactam A showed to be a remarkably strong and selective α3 glycine-gated chloride channel receptor (GlyR) potentiator and a selective α1 GlyR potentiator, respectively [67]. The authors attributed to the glycinyl-lactam moiety the optimal potentiating effect. GlyRs play an essential role coordinating the inhibitory neurotransmission in the spinal cord, brainstem and retina [68].

Using a metabolomics-chemometrics approach, Ali and colleagues [69] identified the metabolites halisulphates 1, 3-5 and suvanine from *Psammocinia* and *Sarcotragus* spp. (Weno Island, Federated States of Micronesia). Among these compounds, halisulphate-5 was the most active substance in the adenosine receptor binding activity [69]. The adenosine A1 receptor has been targeted as an anti-obesity drug, because it blocks the receptor by an antagonist or inverse agonist [70,71].

In this section, the enormous amount of terpenoids and terpenoid-quinones retrieved from Irciniidae species as well as their broad spectrum of activities were revealed. However, none of these studies

discussed the possibility of these compounds to be produced by sponge symbionts. The capacity of marine microorganisms to synthesize these molecules is well documented [15,72–75]. Considering the high diversity of bacterial phyla associated with *Ircinia* spp. and *S. spinosulus* [76–80], it is likely that some of these compounds are indeed produced by the bacterial associates. Further research is thus needed to determine the real producers of the terpenoids and terpenoid-quinones detected in Irciniidae species. Novel insights into this can be gained by the combined use of metagenomics, single-cell genomics, metabolomics and alternative culturing approaches to the microbiome of irciniids.

2. Polyketides

Polyketides comprise the class of compounds for which most compelling evidence exists for the prokaryotic origin of bioactive metabolites in marine sponges [2,81,82]. Displaying diverse structures and bioactivities, polyketides present a worldwide pattern of occurrence across a vast range of marine sponge species.

From an Australian *I. ramosa* 73-deoxychondropsin A was retrieved, whereas chondropsin C was isolated from *Ircinia* sp. collected in the Philippines. These compounds belong to the condropsin family of polyketide-derived macrolide lactams and showed cytotoxicity against the LOX (melanoma) and the MOLT-4 (leukaemia) cancer cell lines [83].

Mycalolide A and C (trixazole macrolide) were isolated from *Sarcotragus* sp. [84]. The former was originally obtained from the sponge *Mycale* sp., showing antagonistic activities towards many pathogenic fungi and cytotoxicity against the B-16 melanoma cancer cell line [85]. Tedanolide C was retrieved from *Ircinia* sp. collected at Papua New Guinea, and was shown to be cytotoxic to the HCT-116 (colorectal) cancer cell line and proposed to act as an inhibitor of protein synthesis [86].

The compounds (+)-psymberin (resembling the pederin family of polyketides), (+)-pederin, (−)-variabilin, (−)-psymbamide A, (−)-preswinholide A, and (+)-swinholide A were obtained from *P.* aff. *bulbosa* (Papua New Guinea) [87,88]. Psymberin showed strong cytotoxic activity against the HCT-116 (colorectal) cancer cell line [87]. In a pioneering study, Bewley and colleagues [89] demonstrated that the occurrence of swinholide A isolated from *Theonella swinhoei* was limited to the mixed population of unicellular heterotrophic bacteria. Further, psymberin was also recovered from *Psammocinia* sp. and exhibited outstanding cytotoxicity against diverse human cancer cells lines, for instance MDA-MB-435 and T-47 (breast), HCT-116 (colon), and SK-MEL-5 and -28 (melanoma). Indeed, psymberin might be one of the most prominent candidates for drug development [90]. In the beetle *Paederus fuscipes*, an uncultured bacterium closely related to *Pseudomonas aeruginosa* was suggested to be the producer of pederin [91].

Although it is not possible to determine whether all polyketides recovered from Irciniidae species have a bacterial origin, evidences from other sponge species and hosts [2,3,92] strongly suggest that members of the complex irciniid microbiome may produce many of these compounds.

Supplementary Figure S1 (see the separate file)

Phylogenetic inference of the most abundant bacterial phyla retrieved from irciniids. All bacterial 16S rRNA gene sequences obtained from Irciniidae species publicly available at the National Center for Biotechnology Information (NCBI) database [93] until December 2013 were downloaded.

Sequences' affiliation was assessed using the Ribosomal Database Project II (RDP) classifier tool [94] with the confidence threshold at 80%. Sequences were then aligned using the SINA web aligner [95] according to their phylum level affiliation. Aligned sequences were imported into the modified Silva 16S rRNA gene database version 102, which contained all sponge-derived 16S rRNA gene sequences available in early 2010 [96], using the Arb parsimony interactive tool [97]. Alignments of several sequences with a confidence threshold below 80% were manually checked and corrected when necessary using the Arb alignment window. Once the affiliation of the sequences was confirmed, phylogenetic trees were constructed with sequences obtained from cultivation (S1a) and cloning-and-sequencing (S1b−i) studies. To this end, an appropriate evolutionary model was determined for each phylogenetic tree using MEGA6 [98], and found to be the general-time reversible model (GTR, [99]) with a discrete gamma-distribution of among-site rate variation (Γ_4) and a proportion of invariant sites (I), except for Alphaproteobacteria and Cyanobacteria inferences, in which the invariant sites did not fit. Maximum likelihood analyses were conducted using MEGA6 [98]. Bootstrap values (1000 repetitions) greater than or equal to 75% are shown on tree nodes. Sequences marked in green, fuchsia and blue are from irciniids collected at the Caribbean region, Eastern Atlantic Ocean and Mediterranean Sea, respectively.

Figure S1a. 16S rRNA-based phylogeny of bacteria cultured from irciniids, containing 328 sequences (-ln likelihood: 7108.6128).

Figure S1b. 16S rRNA-based phylogeny of irciniids-associated Alphaproteobacteria, containing 142 sequences (-ln likelihood: 7635.5613).

Figure S1c. 16S rRNA-based phylogeny of irciniids-associated Gammaproteobacteria, containing 180 sequences (-ln likelihood: 6018.4595).

Figure S1d. 16S rRNA-based phylogeny of irciniids-associated Deltaproteobacteria, containing 136 sequences (-ln likelihood: 4391.2552).

Figure S1e. 16S rRNA-based phylogeny of irciniids-associated Cyanobacteria, containing 200 sequences (-ln likelihood: 1789.9077).

Figure S1f. 16S rRNA-based phylogeny of irciniids-associated Chloroflexi, containing 121 sequences (-ln likelihood: 7709.7985).

Figure S1g. 16S rRNA-based phylogeny of irciniids-associated Acidobacteria, containing 107 sequences (-ln likelihood: 4027.1615).

Figure S1h. 16S rRNA-based phylogeny of irciniids-associated Actinobacteria, containing 41 sequences (-ln likelihood: 3234.7474).

Figure S1i. 16S rRNA-based phylogeny of irciniids-associated Bacteroidetes, containing 50 sequences (-ln likelihood: 4511.6255).

References

1. Taylor, M.W.; Radax, R.; Steger, D.; Wagner, M. Sponge-associated microorganisms: Evolution, ecology, and biotechnological potential. *Microbiol. Mol. Biol. Rev.* **2007**, *71*, 295–347.

2. Piel, J.; Hui, D.Q.; Wen, G.P.; Butzke, D.; Platzer, M.; Fusetani, N.; Matsunaga, S. Antitumor polyketide biosynthesis by an uncultivated bacterial symbiont of the marine sponge *Theonella swinhoei*.. *Proc Natl Acad Sci USA* **2004**, *101*, 16222–16227.
3. Piel, J. Metabolites from symbiotic bacteria. *Nat. Prod. Rep.* **2004**, *21*, 519–538.
4. Kim, T.K.; Fuerst, J.A. Diversity of polyketide synthase genes from bacteria associated with the marine sponge *Pseudoceratina clavata*: Culture-dependent and culture-independent approaches. *Environ. Microbiol.* **2006**, *8*, 1460–1470.
5. Thomas, T.R.A.; Kavlekar, D.P.; LokaBharathi, P.A. Marine drugs from sponge-microbe association: A review. *Mar. Drugs* **2010**, *8*, 1417–1468.
6. Leal, M.C.; Puga, J.; Serodio, J.; Gomes, N.C.M.; Calado, R. Trends in the discovery of new marine natural products from invertebrates over the last two decades—Where and what are we bioprospecting? *PLoS One* **2012**, *7*, e30580.
7. International Union of Pure and Applied Chemistry (IUPAC). Available online: http://www.chem.qmul.ac.uk/iupac/class/terp.html#15 (accessed on 19 July 2013).
8. Wang, G.; Tang, W.; Bidigare, R. Terpenoids as therapeutic drugs and pharmaceutical agents. In *Natural Products: Drug Discovery and Therapeutic Medicine*; Zhang, L., Demain, A.L., Eds.; Humana Press: Totowa, NJ, USA, 2005; pp. 197–227.
9. Motohashi, K.; Ueno, R.; Sue, M.; Furihata, K.; Matsumoto, T.; Dairi, T.; Omura, S.; Seto, H. Studies on terpenoids produced by *Actinomycetes*: Oxaloterpins A, B, C, D, and E, diterpenes from *Streptomyces* sp. KO-3988. *J. Nat. Prod.* **2007**, *70*, 1712–1717.
10. ClinicalTrials.gov. Available online: http://www.clinicaltrials.gov/ct2/results?term=paclitaxel (accessed on 25 July 2013).
11. Sunassee, S.N.; Davies-Coleman, M.T. Cytotoxic and antioxidant marine prenylated quinones and hydroquinones. *Nat. Prod. Rep.* **2012**, *29*, 513–535.
12. Faulkner, D.J. Marine natural products. *Nat. Prod. Rep.* **2001**, *18*, 1–49.
13. Blunt, J.W.; Copp, B.R.; Hu, W.P.; Munro, M.H.G.; Northcote, P.T.; Prinsep, M.R. Marine natural products. *Nat. Prod. Rep.* **2008**, *25*, 35–94.
14. Blunt, J.W.; Copp, B.R.; Hu, W.P.; Munro, M.H.G.; Northcote, P.T.; Prinsep, M.R. Marine natural products. *Nat. Prod. Rep.* **2009**, *26*, 170–244.
15. Blunt, J.W.; Copp, B.R.; Munro, M.H.G.; Northcote, P.T.; Prinsep, M.R. Marine natural products. *Nat. Prod. Rep.* **2010**, *27*, 165–237.
16. Gordaliza, M. Cytotoxic terpene quinones from marine sponges. *Mar. Drugs* **2010**, *8*, 2849–2870.
17. Blunt, J.W.; Copp, B.R.; Munro, M.H.G.; Northcote, P.T.; Prinsep, M.R. Marine natural products. *Nat. Prod. Rep.* **2011**, *28*, 196–268.
18. Solecka, J.; Zajko, J.; Postek, M.; Rajnisz, A. Biologically active secondary metabolites from *Actinomycetes*. *Cent. Eur. J. Biol.* **2012**, *7*, 373–390.
19. Bifulco, G.; Bruno, I.; Minale, L.; Riccio, R.; Debitus, C.; Bourdy, G.; Vassas, A.; Lavayre, J. Bioactive prenylhydroquinone sulfates and a novel C_{31} furanoterpene alcohol sulfate from the marine sponge, *Ircinia* sp. *J. Nat. Prod.* **1995**, *58*, 1444–1449.
20. Loya, S.; Rudi, A.; Kashman, Y.; Hizi, A. Mode of inhibition of HIV reverse transcriptase by 2-hexaprenyl-hydroquinone, a novel general inhibitor of RNA- and DNA-directed DNA polymerases. *Biochem. J.* **1997**, *324*, 721–727.

21. Barrow, C.; Blunt, J.; Munro, M.; Perry, N. Variabilin and related-compounds from a sponge of the genus *Sarcotragus*. *J. Nat. Prod.* **1988**, *51*, 275–281.
22. Rothberg, I.; Shubiak, P. Structure of some antibiotics from sponge *Ircinia strobilina*. *Tetrahedron Lett.* **1975**, 769–772.
23. Martinez, A.; Duque, C.; Sato, N.; Fujimoto, Y. (8*Z*,13*Z*,20*Z*)-strobilinin and (7*Z*,13*Z*,20*Z*)-felixinin: New furanosesterterpene tetronic acids from marine sponges of the genus *Ircinia*. *Chem. Pharm. Bull.* **1997**, *45*, 181–184.
24. Garcia, M.; Rodriguez, A. Palominin, a novel furanosesterterpene from a Caribbean sponge *Ircinia* sp. *Tetrahedron* **1990**, *46*, 1119–1124.
25. Tsoukatou, M.; Hellio, C.; Vagias, C.; Harvala, C.; Roussis, V. Chemical defense and antifouling activity of three Mediterranean sponges of the genus *Ircinia*. *Z. Naturforsch. C* **2002**, *57*, 161–171.
26. Wright, A.; McCarthy, P.; Schulte, G. Sulfircin—A new sesterterpene sulfate from a deep-water sponge of the genus *Ircinia*. *J. Org. Chem.* **1989**, *54*, 3472–3474.
27. Degiulio, A.; Derosa, S.; Divincenzo, G.; Strazzullo, G.; Zavodnik, N. Norsesterterpenes from the north Adriatic sponge *Ircinia oros*. *J. Nat. Prod.* **1990**, *53*, 1503–1507.
28. De Rosa, S.; DeGiulio, A.; Crispino, A.; Iodice, C.; Tommonaro, G. Palinurin and fasciculatin sulfates from two thyrrenean sponges of the genus *Ircinia*. *Nat. Prod. Lett.* **1997**, *10*, 7–12.
29. He, W.H.; Lin, X.P.; Xu, T.H.; Jung, J.H.; Yin, H.; Yang, B.; Liu, Y.H. A new norsesterterpenoid from the sponge species *Sarcotragus*. *Chem. Nat. Compd.* **2012**, *48*, 208–210.
30. De Rosa, S.; Crispino, A.; Degiulio, A.; Iodice, C.; Milone, A. Sulfated polyprenylhydroquinones from the sponge *Ircinia spinosula*. *Nat. Prod. Rep.* **1995**, *58*, 1450–1454.
31. Hellio, C.; Tsoukatou, M.; Maréchal, J.; Aldred, N.; Beaupoil, C.; Clare, A.; Vagias, C.; Roussis, V. Inhibitory effects of Mediterranean sponge extracts and metabolites on larval settlement of the barnacle *Balanus amphitrite*. *Mar. Biotechnol.* **2005**, *7*, 297–305.
32. Tziveleka, L.; Kourounakis, A.; Kourounakis, P.; Roussis, V.; Vagias, C. Antioxidant potential of natural and synthesised polyprenylated hydroquinones. *Bioorg. Med. Chem.* **2002**, *10*, 935–939.
33. Alfano, G.; Cimino, G.; Destefano, S. Palinurin, a new linear sesterterpene from a marine sponge. *Experientia* **1979**, *35*, 1136–1137.
34. Martí, R.; Fontana, A.; Uriz, M.; Cimino, G. Quantitative assessment of natural toxicity in sponges: Toxicity bioassay *versus* compound quantification. *J. Chem. Ecol.* **2003**, *29*, 1307–1318.
35. Choi, K.; Hong, J.; Lee, C.; Kim, D.; Sim, C.; Im, K.; Jung, J. Cytotoxic furanosesterterpenes from a marine sponge *Psammocinia* sp. *J. Nat. Prod.* **2004**, *67*, 1186–1189.
36. Epifanio, R.; Gabriel, R.; Martins, D.; Muricy, G. The sesterterpene variabilin as a fish-predation deterrent in the western Atlantic sponge *Ircinia strobilina*. *J. Chem. Ecol.* **1999**, *25*, 2247–2254.
37. Pawlik, J.; McFall, G.; Zea, S. Does the odor from sponges of the genus *Ircinia* protect them from fish predators? *J. Chem. Ecol.* **2002**, *28*, 1103–1115.
38. Cafieri, F.; Santacro.C; Fattorus.E; Minale, L. Fasciculatin, a novel sesterterpene from sponge *Ircinia fasciculata*. *Tetrahedron* **1972**, *28*, 1579–1583.
39. Rifai, S.; Fassouane, A.; Pinho, P.; Kijjoa, A.; Nazareth, N.; Nascimento, M.; Herz, W. Cytotoxicity and inhibition of lymphocyte proliferation of fasciculatin, a linear furanosesterterpene isolated from *Ircinia variabilis* collected from the Atlantic coast of Morocco. *Mar. Drugs* **2005**, *3*, 15–21.

40. Manes, L.; Naylor, S.; Crews, P.; Bakus, G. Suvanine, a novel sesterterpene from an *Ircinia* marine sponge. *J. Org. Chem.* **1985**, *50*, 284–286.
41. Freeman, C.J.; Gleason, D.F. Chemical defenses, nutritional quality, and structural components in three sponge species: *Ircinia felix*, *I. campana*, and *Aplysina fulva*. *Mar. Biol.* **2010**, *157*, 1083–1093.
42. Freeman, C.J.; Gleason, D.F. Does concentrating chemical defenses within specific regions of marine sponges result in enhanced protection from predators? *Hydrobiologia* **2012**, *687*, 289–297.
43. Liu, Y.; Bae, B.; Alam, N.; Hong, J.; Sim, C.; Lee, C.; Im, K.; Jung, J. New cytotoxic sesterterpenes from the sponge *Sarcotragus* species. *J. Nat. Prod.* **2001**, *64*, 1301–1304.
44. Liu, Y.; Lee, C.; Hong, J.; Jung, J. Cyclitol derivatives from the sponge *Sarcotragus* species. *Bull. Korean Chem. Soc.* **2002**, *23*, 1467–1469.
45. Liu, Y.; Hong, J.; Lee, C.; Im, K.; Kim, N.; Choi, J.; Jung, J. Cytotoxic pyrrolo- and furanoterpenoids from the sponge *Sarcotragus* species. *J. Nat. Prod.* **2002**, *65*, 1307–1314.
46. Liu, Y.; Mansoor, T.; Hong, J.; Lee, C.; Sim, C.; Im, K.; Kim, N.; Jung, J. New cytotoxic sesterterpenoids and norsesterterpenoids from two sponges of the genus *Sarcotragus*. *J. Nat. Prod.* **2003**, *66*, 1451–1456.
47. Liu, Y.; Jung, J.; Zhang, S. Linear pyrrolosesterterpenes from a sponge *Sarcotragus* species. *Biochem. Syst. Ecol.* **2006**, *34*, 774–776.
48. Shin, J.; Rho, J.; See, Y.; Lee, H.; Cho, K.; Sim, C. Sarcotragins A and B, new sesterterpenoid alkaloids from the sponge *Sarcotragus* sp. *Tetrahedron Lett.* **2001**, *42*, 3005–3007.
49. Choi, H.; Choi, Y.; Yee, S.; Im, E.; Jung, J.; Kim, N. Ircinin-1 induces cell cycle arrest and apoptosis in SK-MEL-2 human melanoma cells. *Mol. Carcinog.* **2005**, *44*, 162–173.
50. Kokubo, S.; Yogi, K.; Uddin, M.; Inuzuka, T.; Suenaga, K.; Ueda, K.; Uemura, D. Kohamaic acids A and B, novel cytotoxic sesterterpenic acids, from the marine sponge *Ircinia* sp. *Chem. Lett.* **2001**, *32*, 176–177.
51. Su, J.H.; Tseng, S.W.; Lu, M.C.; Liu, L.L.; Chou, Y.L.; Sung, P.J. Cytotoxic C_{21} and C_{22} terpenoid-derived metabolites from the sponge *Ircinia* sp. *J. Nat. Prod.* **2011**, *74*, 2005–2009.
52. Abed, C.; Legrave, N.; Dufies, M.; Robert, G.; Guerineau, V.; Vacelet, J.; Auberger, P.; Amade, P.; Mehiri, M. A new hydroxylated nonaprenylhydroquinone from the Mediterranean marine sponge *Sarcotragus spinosulus*. *Mar. Drugs* **2011**, *9*, 1210–1219.
53. Shen, Y.; Lo, K.; Lin, Y.; Khalil, A.; Kuo, Y.; Shih, P. Novel linear C_{22}-sesterterpenoids from sponge *Ircinia formosana*. *Tetrahedron Lett.* **2006**, *47*, 4007–4010.
54. Wätjen, W.; Putz, A.; Chovolou, Y.; Kampkotter, A.; Totzke, F.; Kubbutat, M.; Proksch, P.; Konuklugil, B. Hexa-, hepta- and nonaprenylhydroquinones isolated from marine sponges *Sarcotragus muscarum* and *Ircinia fasciculata* inhibit NF-κB signalling in H4IIE cells. *J. Pharm. Pharmacol.* **2009**, *61*, 919–924.
55. Hahn, D.; Won, D.H.; Mun, B.; Kim, H.; Han, C.; Wang, W.; Chun, T.; Park, S.; Yoon, D.; Choi, H.; *et al.* Cytotoxic scalarane sesterterpenes from a Korean marine sponge *Psammocinia* sp. *Bioorg. Med. Chem. Lett.* **2013**, *23*, 2336–2339.
56. Gil, B.; Sanz, M.; Terencio, M.; Degiulio, A.; Derosa, S.; Alcaraz, M.; Paya, M. Effects of marine 2-polyprenyl-1,4-hydroquinones on phospholipase A_2 activity and some inflammatory responses. *Eur. J. Pharmacol.* **1995**, *285*, 281–288.

57. Wakimoto, T.; Maruyama, A.; Matsunaga, S.; Fusetani, N.; Shinoda, K.; Murphy, P. Octa- and nonaprenylhydroquinone sulfates, inhibitors of alpha 1,3-fucosyltransferase VII, from an Australian marine sponge *Sarcotragus* sp. *Bioorg. Med. Chem. Lett.* **1999**, *9*, 727–730.
58. Yang, S.; Chan, T.; Pomponi, S.; Gonsiorek, W.; Chen, G.; Wright, A.; Hipkin, W.; Patel, M.; Gullo, V.; Pramanik, B., *et al.* A new sesterterpene, Sch 599473, from a marine sponge, *Ircinia* sp. *J. Antibiot.* **2003**, *56*, 783–786.
59. Tziveleka, L.; Abatis, D.; Paulus, K.; Bauer, R.; Vagias, C.; Roussis, V. Marine polyprenylated hydroquinones, quinones, and chromenols with inhibitory effects on leukotriene formation. *Chem. Biodivers*. **2005**, *2*, 901–909.
60. Buchanan, M.; Edser, A.; King, G.; Whitmore, J.; Quinn, R. Cheilanthane sesterterpenes, protein kinase inhibitors, from a marine sponge of the genus *Ircinia*. *J. Nat. Prod.* **2001**, *64*, 300–303.
61. Stonik, V.; Makarieva, T.; Dmitrenok, A. Sarcochromenol sulfate A–C and sarcohydroquinone sulfate A−C, new natural-products from the sponge *Sarcotragus spinulosus*. *J. Nat. Prod.* **1992**, *55*, 1256–1260.
62. Takikawa, H.; Kamatani, N.; Nakanishi, K.; Tashiro, T.; Sasaki, M.; Yoshida, H.; Mizushina, Y. Synthetic studies on kohamaic acids: Synthesis of structurally simplified analogs of kohamaic acid A. *Biosci. Biotechnol. Biochem.* **2008**, *72*, 3071–3074.
63. Aktas, N.; Genc, Y.; Gozcelioglu, B.; Konuklugil, B.; Harput, U.S. Radical scavenging effect of different marine sponges from Mediterranean coasts. *Rec. Nat. Prod.* **2013**, *7*, 96–104.
64. Shen, Y.; Shih, P.; Lin, Y.; Lin, Y.; Kuo, Y.; Koo, Y.; Khalil, A. Irciformonins E−K, C_{22}-trinorsesterterpenoids from the sponge *Ircinia formosana*. *Helv. Chim. Acta* **2009**, 2101–2110.
65. Cichewicz, R.; Kenyon, V.; Whitman, S.; Morales, N.; Arguello, J.; Holman, T.; Crews, P. Redox inactivation of human 15-lipoxygenase by marine-derived meroditerpenes and synthetic chromanes: Archetypes for a unique class of selective and recyclable inhibitors. *J. Am. Chem. Soc.* **2004**, *126*, 14910–14920.
66. Balansa, W.; Islam, R.; Fontaine, F.; Piggott, A.M.; Zhang, H.; Webb, T.I.; Gilbert, D.F.; Lynch, J.W.; Capon, R.J. Ircinialactams: Subunit-selective glycine receptor modulators from Australian sponges of the family Irciniidae. *Bioorg. Med. Chem.* **2010**, *18*, 2912–2919.
67. Balansa, W.; Islam, R.; Fontaine, F.; Piggott, A.M.; Zhang, H.; Xiao, X.; Webb, T.L.; Gilbert, D.F.; Lynch, J.W.; Capon, R.J. Sesterterpene glycinyl-lactams: A new class of glycine receptor modulator from Australian marine sponges of the genus *Psammocinia*. *Org. Biomol. Chem.* **2013**, *11*, 4695–4701.
68. Lynch, J.W. Molecular structure and function of the glycine receptor chloride channel. *Physiol. Rev.* **2004**, *84*, 1051–1095.
69. Ali, K.; Iqbal, M.; Yuliana, N.D.; Lee, Y.J.; Park, S.; Han, S.; Lee, J.W.; Lee, H.S.; Verpoorte, R.; Choi, Y.H. Identification of bioactive metabolites against adenosine A1 receptor using NMR-based metabolomics. *Metabolomics* **2013**, *9*, 778–785.
70. Barakat, H.; Davis, J.; Lang, D.; Mustafa, S.J.; McConnaughey, M.M. Differences in the expression of the adenosine A1 receptor in adipose tissue of obese black and white women. *J. Clin. Endocrinol. Metable* **2006**, *91*, 1882–1886.

71. JohanssonA, S.M.; Lindgren, E.; Yang, J.N.; Herling, A.W.; Fredholm, B.B. Adenosine A1 receptors regulate lipolysis and lipogenesis in mouse adipose tissue—Interactions with insulin. *Eur. J. Pharmacol.* **2008**, *597*, 92–101.
72. Mincer, T.J.; Spyere, A.; Jensen, P.R.; Fenical, W. Phylogenetic analyses and diterpenoid production by marine bacteria of the genus *Saprospira*. *Curr. Microbiol.* **2004**, *49*, 300–307.
73. Spyere, A.; Rowley, D.C.; Jensen, P.R.; Fenical, W. New neoverrucosane diterpenoids produced by the marine gliding bacterium *Saprospira grandis*. *J. Nat. Prod.* **2003**, *66*, 818–822.
74. Fenical, W.; Jensen, P.R. Developing a new resource for drug discovery: Marine *Actinomycete* bacteria. *Nat. Chem. Biol.* **2006**, *2*, 666–673.
75. Williams, P.G. Panning for chemical gold: Marine bacteria as a source of new therapeutics. *Trends Biotechnol.* **2009**, *27*, 45–52.
76. Webster, N.S.; Taylor, M.W.; Behnam, F.; Lucker, S.; Rattei, T.; Whalan, S.; Horn, M.; Wagner, M. Deep sequencing reveals exceptional diversity and modes of transmission for bacterial sponge symbionts. *Environ. Microbiol.* **2010**, *12*, 2070–2082.
77. Schmitt, S.; Tsai, P.; Bell, J.; Fromont, J.; Ilan, M.; Lindquist, N.; Perez, T.; Rodrigo, A.; Schupp, P.J.; Vacelet, J.; *et al.* Assessing the complex sponge microbiota: Core, variable and species-specific bacterial communities in marine sponges. *ISME J.* **2012**, *6*, 564–576.
78. Hardoim, C.C.P.; Esteves, A.I.S.; Pires, F.R.; Gonçalves, J.M.S.; Cox, C.J.; Xavier, J.R.; Costa, R. Phylogenetically and spatially close marine sponges harbour divergent bacterial communities. *PLoS One* **2012**, *7*, e53029.
79. Hardoim, C.C.P.; Cardinale, M.; Cúcio, A.; Esteves, A.I.S.; Berg, G.; Xavier, J.R.; Cox, C.; Costa, R. Bacterial cultivation bias examined for keratose marine sponges. Unpublised work, 2014.
80. Hardoim, C.C.P.; Costa, R. Temporal dynamics of prokaryotic communities in the marine sponge *Sarcotragus spinosulus*. *Mol. Ecol.* **2014**. *23*, 3097–3112.
81. Fisch, K.; Gurgui, C.; Heycke, N.; van der Sar, S.; Anderson, S.; Webb, V.; Taudien, S.; Platzer, M.; Rubio, B.; Robinson, S., *et al.* Polyketide assembly lines of uncultivated sponge symbionts from structure-based gene targeting. *Nat. Chem. Biol.* **2009**, *5*, 494–501.
82. Hentschel, U.; Piel, J.; Degnan, S.M.; Taylor, M.W. Genomic insights into the marine sponge microbiome. *Nat. Rev. Microbiol.* **2012**, *10*, 641–654.
83. Rashid, M.; Gustafson, K.; Boyd, M. New chondropsin macrolide lactams from marine sponges in the genus *Ircinia*. *Tetrahedron Lett.* **2001**, *42*, 1623–1626.
84. Liu, Y.; Ji, H.; Zhang, S.; Jung, J.; Xu, T. Trisoxazole macrolides from the sponge *Sarcotragus* species. *Chem. Nat. Compd.* **2008**, *44*, 140–141.
85. Fusetani, N.; Yasumuro, K.; Matsunaga, S.; Hashimoto, K. Mycalolides-A−C, hybrid macrolides of ulapualides and halichondramide, from a sponge of the genus *Mycale*. *Tetrahedron Lett.* **1989**, *30*, 2809–2812.
86. Chevallier, C.; Bugni, T.; Feng, X.; Harper, M.; Orendt, A.; Ireland, C. Tedanolide C: A potent new 18-membered-ring cytotoxic macrolide isolated from the Papua New Guinea marine sponge *Ircinia* sp. *J. Org. Chem.* **2006**, *71*, 2510–2513.
87. Robinson, S.; Tenney, K.; Yee, D.; Martinez, L.; Media, J.; Valeriote, F.; van Soest, R.; Crews, P. Probing the bioactive constituents from chemotypes of the sponge *Psammocinia* aff. *bulbosa*. *J. Nat. Prod.* **2007**, *70*, 1002–1009.

88. Rubio, B.; Robinson, S.; Avalos, C.; Valeriote, F.; de Voogd, N.; Crews, P. Revisiting the sponge sources, stereostructure, and biological activity of cyclocinamide A. *J. Nat. Prod.* **2008**, *71*, 1475–1478.
89. Bewley, C.A.; Holland, N.D.; Faulkner, D.J. Two classes of metabolites from *Theonella swinhoei* are localized in distinct populations of bacterial symbionts. *Experientia* **1996**, *52*, 716–722.
90. Cichewicz, R.; Valeriote, F.; Crews, P. Psymberin, a potent sponge-derived cytotoxin from *Psammocinia* distantly related to the pederin family. *Org. Lett.* **2004**, *6*, 1951–1954.
91. Piel, J.; Hofer, I.; Hui, D.Q. Evidence for a symbiosis island involved in horizontal acquisition of pederin biosynthetic capabilities by the bacterial symbiont of *Paederus fuscipes* beetles. *J. Bacteriol.* **2004,** *186,* 1280–1286.
92. Piel, J.; Butzke, D.; Fusetani, N.; Hui, D.Q.; Platzer, M.; Wen, G.P.; Matsunaga, S. Exploring the chemistry of uncultivated bacterial symbionts: Antitumor polyketides of the pederin family. *J. Nat. Prod.* **2005**, *68*, 472–479.
93. Altschul, S.F.; Madden, T.L.; Schaffer, A.A.; Zhang, J.H.; Zhang, Z.; Miller, W.; Lipman, D.J. Gapped blast and psi-blast: A new generation of protein database search programs. *Nucl. Acids Res.* **1997**, *25*, 3389–3402.
94. Wang, Q.; Garrity, G.M.; Tiedje, J.M.; Cole, J.R. Naïve bayesian classifier for rapid assignment of rRNA sequences into the new bacterial taxonomy. *Appl. Environ. Microbiol.* **2007**, *73,* 5261–5267.
95. Pruesse, E.; Quast, C.; Knittel, K.; Fuchs, B.M.; Ludwig, W.G.; Peplies, J.; Glockner, F.O. Silva: A comprehensive online resource for quality checked and aligned ribosomal RNA sequence data compatible with Arb. *Nucl. Acids Res.* **2007**, *35,* 7188–7196.
96. Simister, R.L.; Deines, P.; Botte, E.S.; Webster, N.S.; Taylor, M.W. Sponge-specific clusters revisited: A comprehensive phylogeny of sponge-associated microorganisms. *Environ. Microbiol.* **2012**, *14,* 517–524.
97. Ludwig, W.; Strunk, O.; Westram, R.; Richter, L.; Meier, H.; Yadhukumar; Buchner, A.; Lai, T.; Steppi, S.; Jobb, G.; *et al.* Arb: A software environment for sequence data. *Nucl. Acids Res.* **2004**, *32*, 1363–1371.
98. Tamura, K.; Stecher, G.; Peterson, D.; Filipski, A.; Kumar, S. Mega6: Molecular evolutionary genetics analysis version 6.0. *Mol. Biol. Evol.* **2011**, *30*, 2726–2729.
99. Rodriguez, F.; Oliver, J.L.; Marin, A.; Medina, J.R. The general stochastic-model of nucleotide substitution. *J. Theor. Biol.* **1990**, *142*, 485–501.

© 2014 by the authors; licensee MDPI, Basel, Switzerland. This article is an open access article distributed under the terms and conditions of the Creative Commons Attribution license (http://creativecommons.org/licenses/by/3.0/).
