# Supplementary material for: Microbial Communities and Bioactive Compounds in Marine Sponges of the Family Irciniidae—A Review
Source: Mar Drugs. 2014 Sep 30;12(10):5089–122. doi: 10.3390/md12105089 (PMC4210886; doi:10.3390/md12105089)
Supplement: Supplementary File 2 [file marinedrugs-12-05089-s002.pdf]

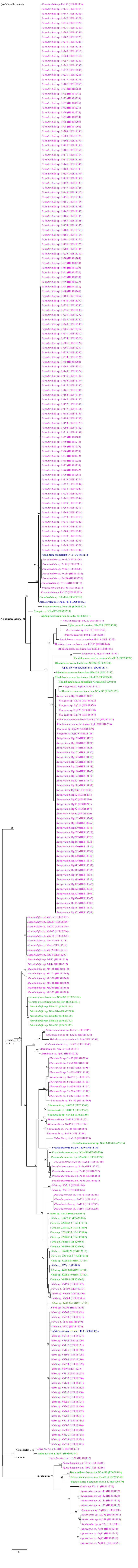
(a) Culturable bacteria
0.1

(b) Alphaproteobacteria

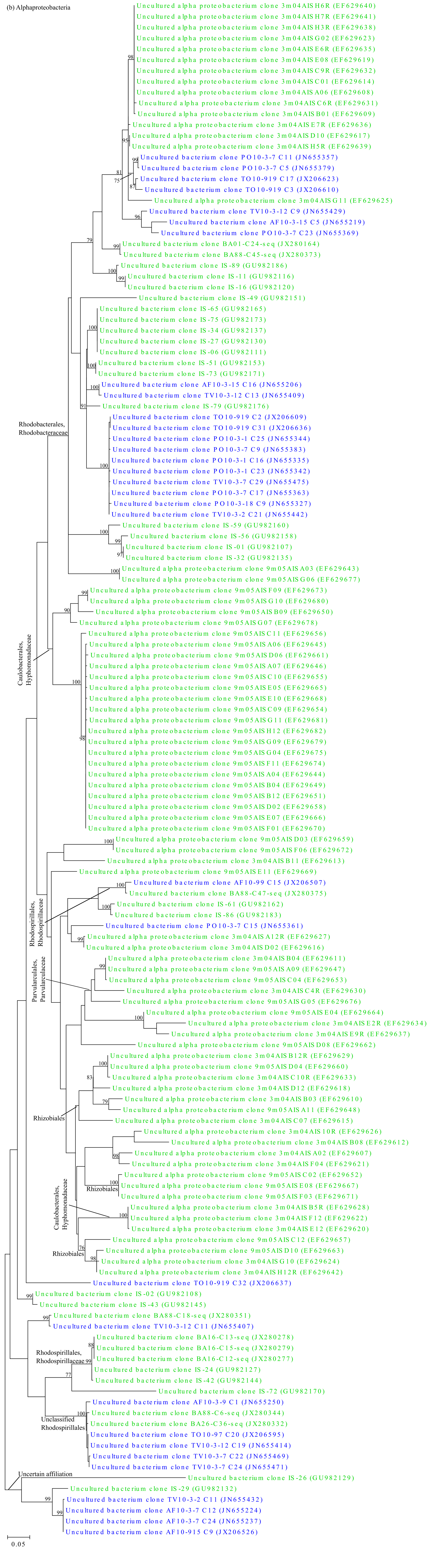

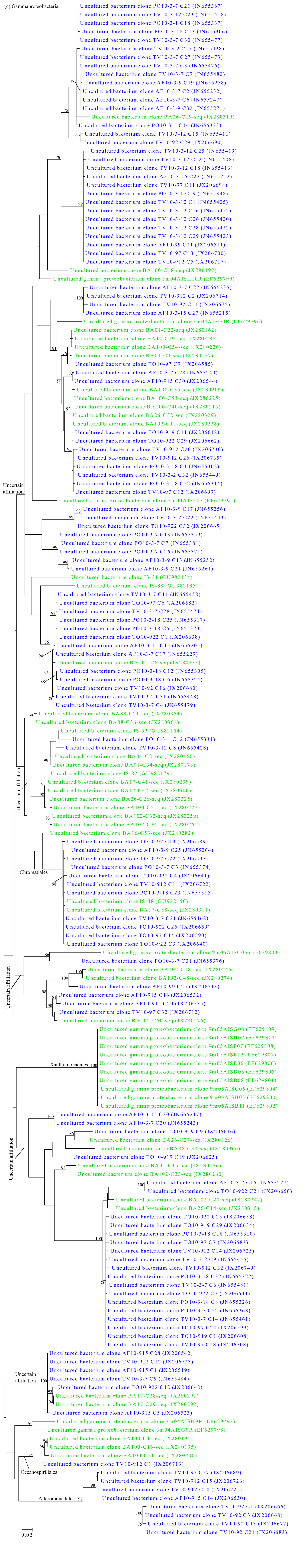
(c) Gammaproteobacteria
Uncultured bacterium clone PO10-3-7 C21 (JN655367)
Uncultured bacterium clone TV10-3-12 C23 (JN655418)
Uncultured bacterium clone PO10-3-1 C18 (JN655337)
Uncultured bacterium clone PO10-3-18 C13 (JN655306)
Uncultured bacterium clone TV10-3-7 C30 (JN655477)
Uncultured bacterium clone TV10-3-2 C17 (JN655438)
Uncultured bacterium clone TV10-3-7 C27 (JN655473)
Uncultured bacterium clone TV10-3-7 C3 (JN655476)
Uncultured bacterium clone TV10-3-7 C7 (JN655482)
Uncultured bacterium clone AF10-3-9 C19 (JN655258)
Uncultured bacterium clone AF10-3-7 C2 (JN655232)
Uncultured bacterium clone AF10-3-7 C6 (JN655247)
Uncultured bacterium clone AF10-3-9 C32 (JN655271)
Uncultured bacterium clone BA26-C19-seq (JX280319)
Uncultured bacterium clone PO10-3-1 C14 (JN655333)
Uncultured bacterium clone TV10-3-12 C15 (JN655411)
Uncultured bacterium clone TV10-92 C29 (JX206690)
Uncultured bacterium clone TV10-3-12 C25 (JN655419)
Uncultured bacterium clone TV10-3-12 C12 (JN655408)
Uncultured bacterium clone TV10-3-12 C18 (JN655413)
Uncultured bacterium clone AF10-3-15 C22 (JN655212)
Uncultured bacterium clone TV10-97 C11 (JX206698)
Uncultured bacterium clone PO10-3-1 C19 (JN655338)
Uncultured bacterium clone TV10-3-12 C11 (JN655405)
Uncultured bacterium clone TV10-3-12 C16 (JN655412)
Uncultured bacterium clone TV10-3-12 C26 (JN655420)
Uncultured bacterium clone TV10-3-12 C28 (JN655422)
Uncultured bacterium clone TV10-3-12 C29 (JN655423)
Uncultured bacterium clone AF10-99 C21 (JX206511)
Uncultured bacterium clone TV10-97 C13 (JX206700)
Uncultured bacterium clone TV10-912 C5 (JX206717)
Uncultured bacterium clone BA100-C18-seq (JX280197)
Uncultured gamma proteobacterium clone 3m04AISH10R (EF629799)
Uncultured bacterium clone AF10-3-7 C22 (JN655235)
Uncultured bacterium clone TV10-912 C2 (JX206714)
Uncultured bacterium clone TV10-92 C11 (JX206675)
Uncultured bacterium clone AF10-3-15 C27 (JN655215)
Uncultured gamma proteobacterium clone 3m04AISD4R (EF629796)
Uncultured bacterium clone BA01-C22-seq (JX280162)
Uncultured bacterium clone BA17-C19-seq (JX280288)
Uncultured bacterium clone BA100-C54-seq (JX280226)
Uncultured bacterium clone BA01-C4-seq (JX280177)
Uncultured bacterium clone TO10-97 C9 (JX206585)
Uncultured bacterium clone AF10-3-7 C28 (JN655240)
Uncultured bacterium clone AF10-915 C30 (JX206544)
Uncultured bacterium clone BA100-C35-seq (JX280209)
Uncultured bacterium clone BA100-C53-seq (JX280225)
Uncultured bacterium clone BA100-C40-seq (JX280215)
Uncultured bacterium clone BA26-C32-seq (JX280329)
Uncultured bacterium clone BA102-C11-seq (JX280238)
Uncultured bacterium clone TO10-919 C11 (JX206618)
Uncultured bacterium clone TO10-922 C29 (JX206662)
Uncultured bacterium clone TV10-912 C20 (JX206730)
Uncultured bacterium clone TV10-912 C26 (JX206735)
Uncultured bacterium clone PO10-3-18 C1 (JN655302)
Uncultured bacterium clone TV10-3-2 C32 (JN655449)
Uncultured bacterium clone PO10-3-18 C22 (JN655314)
Uncultured bacterium clone TV10-97 C12 (JX206699)
Uncertain affiliation
Uncultured gamma proteobacterium clone 3m04AISF07 (EF629795)
Uncultured bacterium clone AF10-3-9 C17 (JN655256)
Uncultured bacterium clone TV10-3-2 C22 (JN655443)
Uncultured bacterium clone TO10-922 C32 (JX206665)
Uncultured bacterium clone PO10-3-7 C13 (JN655359)
Uncultured bacterium clone PO10-3-7 C7 (JN655381)
Uncultured bacterium clone PO10-3-7 C26 (JN655371)
Uncultured bacterium clone AF10-3-9 C13 (JN655252)
Uncultured bacterium clone AF10-3-9 C21 (JN655261)
Uncultured bacterium clone IS-31 (GU982134)
Uncultured bacterium clone IS-88 (GU982185)
Uncultured bacterium clone TV10-3-7 C11 (JN655458)
Uncultured bacterium clone TO10-97 C6 (JX206582)
Uncultured bacterium clone TV10-3-7 C28 (JN655474)
Uncultured bacterium clone PO10-3-18 C25 (JN655317)
Uncultured bacterium clone PO10-3-18 C5 (JN655323)
Uncultured bacterium clone TO10-922 C1 (JX206638)
Uncultured bacterium clone AF10-3-15 C15 (JN655205)
Uncultured bacterium clone AF10-3-7 C17 (JN655229)
Uncultured bacterium clone BA102-C6-seq (JX280233)
Uncultured bacterium clone PO10-3-18 C12 (JN655305)
Uncultured bacterium clone PO10-3-18 C6 (JN655324)
Uncultured bacterium clone TV10-92 C16 (JX206680)
Uncultured bacterium clone TV10-3-2 C31 (JN655448)
Uncultured bacterium clone TV10-3-7 C4 (JN655479)
Uncultured bacterium clone BA88-C21-seq (JX280354)
Uncultured bacterium clone BA88-C36-seq (JX280364)
Uncultured bacterium clone IS-52 (GU982154)
Uncultured bacterium clone PO10-3-1 C12 (JN655331)
Uncultured bacterium clone TV10-3-12 C8 (JN655428)
Uncultured bacterium clone BA01-C2-seq (JX280160)
Uncultured bacterium clone BA01-C34-seq (JX280173)
Uncultured bacterium clone IS-82 (GU982179)
Uncultured bacterium clone BA17-C41-seq (JX280299)
Uncultured bacterium clone BA17-C42-seq (JX280300)
Uncultured bacterium clone BA26-C26-seq (JX280325)
Uncultured bacterium clone BA100-C55-seq (JX280227)
Uncultured bacterium clone BA102-C32-seq (JX280259)
Uncultured bacterium clone BA102-C34-seq (JX280261)
Uncertain affiliation
Uncultured bacterium clone BA16-C53-seq (JX280282)
Uncultured bacterium clone TO10-97 C13 (JX206589)
Uncultured bacterium clone AF10-3-9 C25 (JN655264)
Uncultured bacterium clone TO10-97 C22 (JX206597)
Uncultured bacterium clone PO10-3-7 C3 (JN655374)
Uncultured bacterium clone TO10-922 C4 (JX206641)
Uncultured bacterium clone TV10-912 C11 (JX206722)
Uncultured bacterium clone PO10-3-18 C23 (JN655315)
Uncultured bacterium clone IS-48 (GU982150)
Uncultured bacterium clone BA17-C58-seq (JX280311)
Uncultured bacterium clone TV10-3-7 C21 (JN655468)
Uncultured bacterium clone TO10-922 C26 (JX206659)
Uncultured bacterium clone TO10-97 C14 (JX206590)
Uncultured bacterium clone TO10-922 C3 (JX206640)
Chromatiales
Uncultured gamma proteobacterium clone 9m05AISC05 (EF629803)
Uncultured bacterium clone PO10-3-7 C31 (JN655376)
Uncultured bacterium clone BA102-C18-seq (JX280245)
Uncultured bacterium clone BA102-C48-seq (JX280274)
Uncultured bacterium clone AF10-99 C25 (JX206513)
Uncultured bacterium clone AF10-915 C16 (JX206532)
Uncultured bacterium clone AF10-915 C20 (JX206535)
Uncultured bacterium clone TV10-97 C32 (JX206712)
Uncultured bacterium clone BA102-C50-seq (JX280276)
Uncertain affiliation
Uncultured gamma proteobacterium clone 9m05AISG08 (EF629809)
Uncultured gamma proteobacterium clone 9m05AISH07 (EF629810)
Uncultured gamma proteobacterium clone 9m05AISF07 (EF629808)
Uncultured gamma proteobacterium clone 9m05AISE12 (EF629807)
Uncultured gamma proteobacterium clone 9m05AISD06 (EF629806)
Uncultured gamma proteobacterium clone 9m05AISD05 (EF629805)
Uncultured gamma proteobacterium clone 9m05AISB08 (EF629801)
Uncultured gamma proteobacterium clone 9m05AISC06 (EF629804)
Uncultured gamma proteobacterium clone 9m05AISB03 (EF629800)
Uncultured gamma proteobacterium clone 9m05AISB11 (EF629802)
Xanthomonadales
Uncultured bacterium clone AF10-3-15 C30 (JN655217)
Uncultured bacterium clone AF10-3-7 C30 (JN655243)
Uncultured bacterium clone TO10-919 C9 (JX206616)
Uncultured bacterium clone BA26-C27-seq (JX280326)
Uncultured bacterium clone BA88-C38-seq (JX280366)
Uncultured bacterium clone TO10-919 C19 (JX206625)
Uncultured bacterium clone BA01-C15-seq (JX280156)
Uncultured bacterium clone BA102-C33-seq (JX280260)
Uncultured bacterium clone AF10-3-7 C15 (JN655227)
Uncultured bacterium clone TO10-922 C21 (JX206656)
Uncultured bacterium clone BA102-C20-seq (JX280247)
Uncultured bacterium clone BA26-C14-seq (JX280315)
Uncultured bacterium clone TO10-922 C25 (JX206658)
Uncultured bacterium clone TO10-919 C29 (JX206634)
Uncultured bacterium clone PO10-3-18 C18 (JN655310)
Uncultured bacterium clone TO10-97 C7 (JX206583)
Uncultured bacterium clone TV10-912 C14 (JX206725)
Uncultured bacterium clone TV10-3-2 C9 (JN655455)
Uncultured bacterium clone TV10-912 C32 (JX206740)
Uncultured bacterium clone PO10-3-18 C32 (JN655322)
Uncultured bacterium clone TV10-3-7 C6 (JN655481)
Uncultured bacterium clone TO10-922 C7 (JX206644)
Uncultured bacterium clone PO10-3-18 C8 (JN655326)
Uncultured bacterium clone PO10-3-7 C22 (JN655368)
Uncultured bacterium clone TV10-3-7 C14 (JN655461)
Uncultured bacterium clone TO10-97 C24 (JX206599)
Uncultured bacterium clone TO10-919 C1 (JX206608)
Uncultured bacterium clone TV10-97 C28 (JX206708)
Uncertain affiliation
Uncultured bacterium clone AF10-915 C28 (JX206542)
Uncultured bacterium clone TV10-912 C12 (JX206723)
Uncultured bacterium clone AF10-915 C1 (JX206519)
Uncultured bacterium clone TV10-3-7 C9 (JN655484)
Uncultured bacterium clone TO10-922 C12 (JX206648)
Uncultured bacterium clone BA17-C28-seq (JX280291)
Uncultured bacterium clone BA17-C29-seq (JX280292)
Uncultured bacterium clone AF10-915 C5 (JX206523)
Uncertain affiliation
Uncultured gamma proteobacterium clone 3m04AISH9R (EF629797)
Uncultured gamma proteobacterium clone 3m04AISG9R (EF629798)
Uncultured bacterium clone BA100-C1-seq (JX280191)
Uncultured bacterium clone BA100-C16-seq (JX280195)
Uncultured bacterium clone BA100-C21-seq (JX280200)
Uncultured bacterium clone TV10-912 C1 (JX206713)
Oceanospirillales
Uncultured bacterium clone TV10-92 C27 (JX206689)
Uncultured bacterium clone TV10-912 C15 (JX206726)
Uncultured bacterium clone TV10-912 C10 (JX206721)
Uncultured bacterium clone AF10-915 C14 (JX206530)
Alteromonadales
Uncultured bacterium clone TV10-92 C1 (JX206666)
Uncultured bacterium clone TV10-92 C3 (JX206668)
Uncultured bacterium clone TV10-92 C13 (JX206677)
Uncultured bacterium clone TV10-92 C21 (JX206683)
0.02

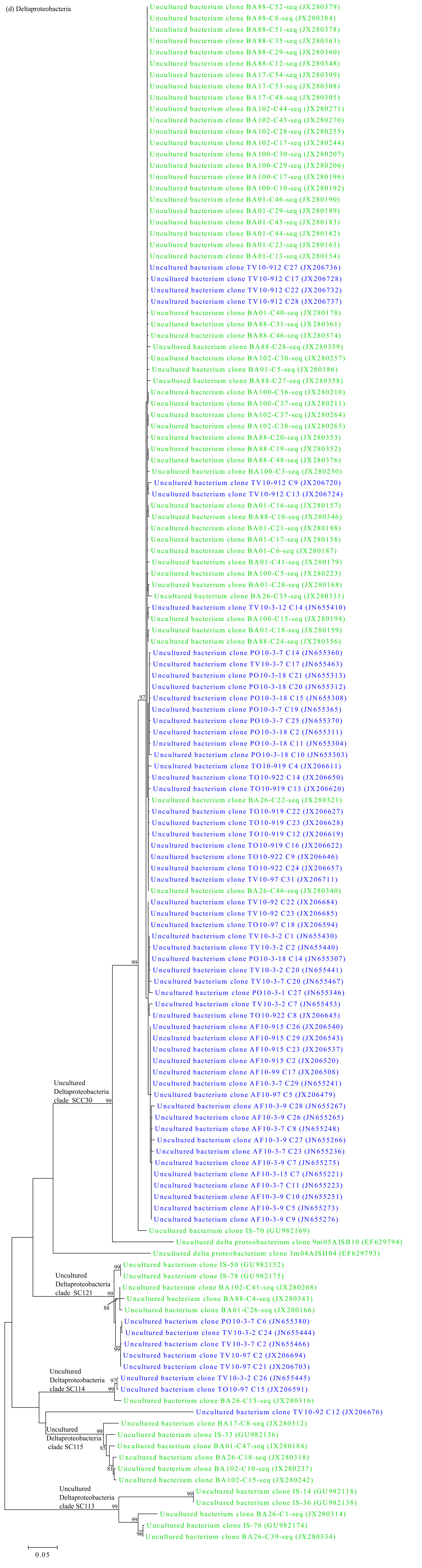

(d) Deltaproteobacteria
Uncultured bacterium clone BA88-C52-seq (JX280379)
Uncultured bacterium clone BA88-C8-seq (JX280384)
Uncultured bacterium clone BA88-C51-seq (JX280378)
Uncultured bacterium clone BA88-C35-seq (JX280363)
Uncultured bacterium clone BA88-C29-seq (JX280360)
Uncultured bacterium clone BA88-C12-seq (JX280348)
Uncultured bacterium clone BA17-C54-seq (JX280309)
Uncultured bacterium clone BA17-C53-seq (JX280308)
Uncultured bacterium clone BA17-C48-seq (JX280305)
Uncultured bacterium clone BA102-C44-seq (JX280271)
Uncultured bacterium clone BA102-C43-seq (JX280270)
Uncultured bacterium clone BA102-C28-seq (JX280255)
Uncultured bacterium clone BA102-C17-seq (JX280244)
Uncultured bacterium clone BA100-C30-seq (JX280207)
Uncultured bacterium clone BA100-C29-seq (JX280206)
Uncultured bacterium clone BA100-C17-seq (JX280196)
Uncultured bacterium clone BA100-C10-seq (JX280192)
Uncultured bacterium clone BA01-C46-seq (JX280190)
Uncultured bacterium clone BA01-C29-seq (JX280189)
Uncultured bacterium clone BA01-C45-seq (JX280183)
Uncultured bacterium clone BA01-C44-seq (JX280182)
Uncultured bacterium clone BA01-C23-seq (JX280163)
Uncultured bacterium clone BA01-C13-seq (JX280154)
Uncultured bacterium clone TV10-912 C27 (JX206736)
Uncultured bacterium clone TV10-912 C17 (JX206728)
Uncultured bacterium clone TV10-912 C22 (JX206732)
Uncultured bacterium clone TV10-912 C28 (JX206737)
Uncultured bacterium clone BA01-C40-seq (JX280178)
Uncultured bacterium clone BA88-C31-seq (JX280361)
Uncultured bacterium clone BA88-C46-seq (JX280374)
Uncultured bacterium clone BA88-C28-seq (JX280359)
Uncultured bacterium clone BA102-C30-seq (JX280257)
Uncultured bacterium clone BA01-C5-seq (JX280186)
Uncultured bacterium clone BA88-C27-seq (JX280358)
Uncultured bacterium clone BA100-C36-seq (JX280210)
Uncultured bacterium clone BA100-C37-seq (JX280211)
Uncultured bacterium clone BA102-C37-seq (JX280264)
Uncultured bacterium clone BA102-C38-seq (JX280265)
Uncultured bacterium clone BA88-C20-seq (JX280353)
Uncultured bacterium clone BA88-C19-seq (JX280352)
Uncultured bacterium clone BA88-C48-seq (JX280376)
Uncultured bacterium clone BA100-C3-seq (JX280230)
Uncultured bacterium clone TV10-912 C9 (JX206720)
Uncultured bacterium clone TV10-912 C13 (JX206724)
Uncultured bacterium clone BA01-C16-seq (JX280157)
Uncultured bacterium clone BA88-C10-seq (JX280346)
Uncultured bacterium clone BA01-C21-seq (JX280188)
Uncultured bacterium clone BA01-C17-seq (JX280158)
Uncultured bacterium clone BA01-C6-seq (JX280187)
Uncultured bacterium clone BA01-C41-seq (JX280179)
Uncultured bacterium clone BA100-C5-seq (JX280223)
Uncultured bacterium clone BA01-C28-seq (JX280168)
Uncultured bacterium clone BA26-C35-seq (JX280331)
Uncultured bacterium clone TV10-3-12 C14 (JN655410)
Uncultured bacterium clone BA100-C15-seq (JX280194)
Uncultured bacterium clone BA01-C18-seq (JX280159)
Uncultured bacterium clone BA88-C24-seq (JX280356)
Uncultured bacterium clone PO10-3-7 C14 (JN655360)
Uncultured bacterium clone TV10-3-7 C17 (JN655463)
Uncultured bacterium clone PO10-3-18 C21 (JN655313)
Uncultured bacterium clone PO10-3-18 C20 (JN655312)
97
Uncultured bacterium clone PO10-3-18 C15 (JN655308)
Uncultured bacterium clone PO10-3-7 C19 (JN655365)
Uncultured bacterium clone PO10-3-7 C25 (JN655370)
Uncultured bacterium clone PO10-3-18 C2 (JN655311)
Uncultured bacterium clone PO10-3-18 C11 (JN655304)
Uncultured bacterium clone PO10-3-18 C10 (JN655303)
Uncultured bacterium clone TO10-919 C4 (JX206611)
Uncultured bacterium clone TO10-922 C14 (JX206650)
Uncultured bacterium clone TO10-919 C13 (JX206620)
Uncultured bacterium clone BA26-C22-seq (JX280321)
Uncultured bacterium clone TO10-919 C22 (JX206627)
Uncultured bacterium clone TO10-919 C23 (JX206628)
Uncultured bacterium clone TO10-919 C12 (JX206619)
Uncultured bacterium clone TO10-919 C16 (JX206622)
Uncultured bacterium clone TO10-922 C9 (JX206646)
Uncultured bacterium clone TO10-922 C24 (JX206657)
Uncultured bacterium clone TV10-97 C31 (JX206711)
Uncultured bacterium clone BA26-C46-seq (JX280340)
Uncultured bacterium clone TV10-92 C22 (JX206684)
Uncultured bacterium clone TV10-92 C23 (JX206685)
Uncultured bacterium clone TO10-97 C18 (JX206594)
Uncultured bacterium clone TV10-3-2 C1 (JN655430)
Uncultured bacterium clone TV10-3-2 C2 (JN655440)
99
Uncultured bacterium clone PO10-3-18 C14 (JN655307)
Uncultured bacterium clone TV10-3-2 C20 (JN655441)
Uncultured bacterium clone TV10-3-7 C20 (JN655467)
Uncultured bacterium clone PO10-3-1 C27 (JN655346)
Uncultured bacterium clone TV10-3-2 C7 (JN655453)
Uncultured bacterium clone TO10-922 C8 (JX206645)
Uncultured bacterium clone AF10-915 C26 (JX206540)
Uncultured bacterium clone AF10-915 C29 (JX206543)
Uncultured bacterium clone AF10-915 C23 (JX206537)
Uncultured bacterium clone AF10-915 C2 (JX206520)
Uncultured bacterium clone AF10-99 C17 (JX206508)
Uncultured bacterium clone AF10-3-7 C29 (JN655241)
Uncultured Deltaproteobacteria clade SCC30
99
Uncultured bacterium clone AF10-97 C5 (JX206479)
Uncultured bacterium clone AF10-3-9 C28 (JN655267)
Uncultured bacterium clone AF10-3-9 C26 (JN655265)
Uncultured bacterium clone AF10-3-7 C8 (JN655248)
Uncultured bacterium clone AF10-3-9 C27 (JN655266)
Uncultured bacterium clone AF10-3-7 C23 (JN655236)
Uncultured bacterium clone AF10-3-9 C7 (JN655275)
Uncultured bacterium clone AF10-3-15 C7 (JN655221)
Uncultured bacterium clone AF10-3-7 C11 (JN655223)
Uncultured bacterium clone AF10-3-9 C10 (JN655251)
Uncultured bacterium clone AF10-3-9 C5 (JN655273)
Uncultured bacterium clone AF10-3-9 C9 (JN655276)
Uncultured bacterium clone IS-70 (GU982169)
Uncultured delta proteobacterium clone 9m05AISB10 (EF629794)
Uncultured delta proteobacterium clone 3m04AISH04 (EF629793)
99
Uncultured bacterium clone IS-50 (GU982152)
Uncultured bacterium clone IS-78 (GU982175)
Uncultured Deltaproteobacteria clade SC121
Uncultured bacterium clone BA102-C41-seq (JX280268)
99
Uncultured bacterium clone BA88-C4-seq (JX280343)
88
Uncultured bacterium clone BA01-C26-seq (JX280166)
Uncultured bacterium clone PO10-3-7 C6 (JN655380)
Uncultured bacterium clone TV10-3-2 C24 (JN655444)
Uncultured bacterium clone TV10-3-7 C2 (JN655466)
99
Uncultured bacterium clone TV10-97 C2 (JX206694)
Uncultured bacterium clone TV10-97 C21 (JX206703)
Uncultured Deltaproteobacteria clade SC114
97
Uncultured bacterium clone TV10-3-2 C26 (JN655445)
99
Uncultured bacterium clone TO10-97 C15 (JX206591)
Uncultured bacterium clone BA26-C15-seq (JX280316)
Uncultured bacterium clone TV10-92 C12 (JX206676)
Uncultured Deltaproteobacteria clade SC115
99
Uncultured bacterium clone BA17-C8-seq (JX280312)
Uncultured bacterium clone IS-33 (GU982136)
85
Uncultured bacterium clone BA01-C47-seq (JX280184)
Uncultured bacterium clone BA26-C18-seq (JX280318)
81
Uncultured bacterium clone BA102-C10-seq (JX280237)
Uncultured bacterium clone BA102-C15-seq (JX280242)
Uncultured Deltaproteobacteria clade SC113
99
Uncultured bacterium clone IS-14 (GU982118)
99
Uncultured bacterium clone IS-36 (GU982138)
99
Uncultured bacterium clone BA26-C1-seq (JX280314)
Uncultured bacterium clone IS-76 (GU982174)
96
Uncultured bacterium clone BA26-C39-seq (JX280334)
0.05

(e) Cyanobacteria

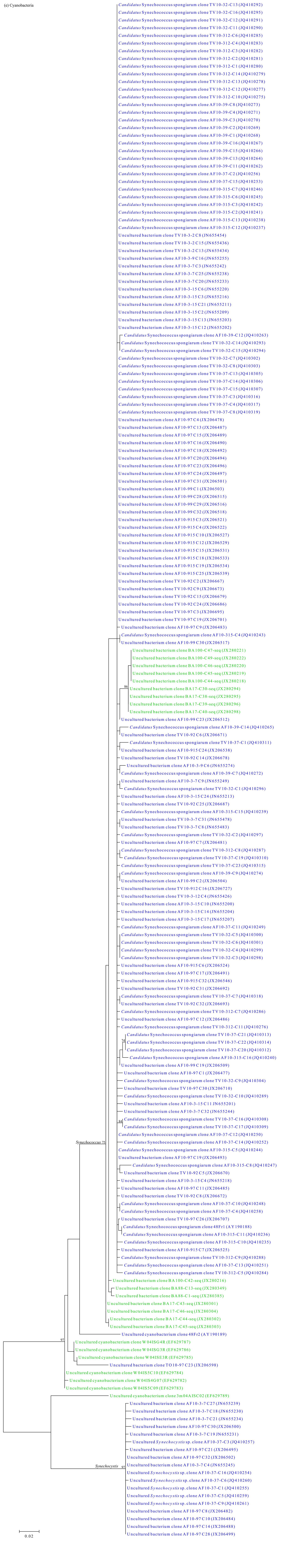

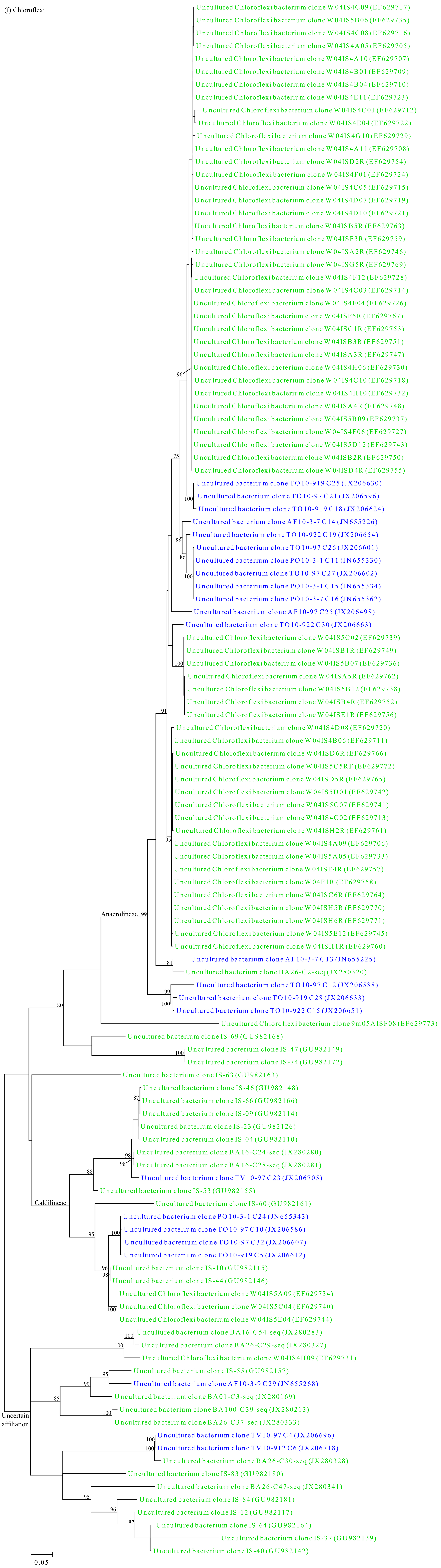
(f) Chloroflexi
Uncultured Chloroflexi bacterium clone W04IS4C09 (EF629717)
Uncultured Chloroflexi bacterium clone W04IS5B06 (EF629735)
Uncultured Chloroflexi bacterium clone W04IS4C08 (EF629716)
Uncultured Chloroflexi bacterium clone W04IS4A05 (EF629705)
Uncultured Chloroflexi bacterium clone W04IS4A10 (EF629707)
Uncultured Chloroflexi bacterium clone W04IS4B01 (EF629709)
Uncultured Chloroflexi bacterium clone W04IS4B04 (EF629710)
Uncultured Chloroflexi bacterium clone W04IS4E11 (EF629723)
Uncultured Chloroflexi bacterium clone W04IS4C01 (EF629712)
Uncultured Chloroflexi bacterium clone W04IS4E04 (EF629722)
Uncultured Chloroflexi bacterium clone W04IS4G10 (EF629729)
Uncultured Chloroflexi bacterium clone W04IS4A11 (EF629708)
Uncultured Chloroflexi bacterium clone W04ISD2R (EF629754)
Uncultured Chloroflexi bacterium clone W04IS4F01 (EF629724)
Uncultured Chloroflexi bacterium clone W04IS4C05 (EF629715)
Uncultured Chloroflexi bacterium clone W04IS4D07 (EF629719)
Uncultured Chloroflexi bacterium clone W04IS4D10 (EF629721)
Uncultured Chloroflexi bacterium clone W04ISB5R (EF629763)
Uncultured Chloroflexi bacterium clone W04ISF3R (EF629759)
Uncultured Chloroflexi bacterium clone W04ISA2R (EF629746)
Uncultured Chloroflexi bacterium clone W04ISG5R (EF629769)
Uncultured Chloroflexi bacterium clone W04IS4F12 (EF629728)
Uncultured Chloroflexi bacterium clone W04IS4C03 (EF629714)
Uncultured Chloroflexi bacterium clone W04IS4F04 (EF629726)
Uncultured Chloroflexi bacterium clone W04ISF5R (EF629767)
Uncultured Chloroflexi bacterium clone W04ISC1R (EF629753)
Uncultured Chloroflexi bacterium clone W04ISB3R (EF629751)
Uncultured Chloroflexi bacterium clone W04ISA3R (EF629747)
Uncultured Chloroflexi bacterium clone W04IS4H06 (EF629730)
Uncultured Chloroflexi bacterium clone W04IS4C10 (EF629718)
Uncultured Chloroflexi bacterium clone W04IS4H10 (EF629732)
Uncultured Chloroflexi bacterium clone W04ISA4R (EF629748)
Uncultured Chloroflexi bacterium clone W04IS5B09 (EF629737)
Uncultured Chloroflexi bacterium clone W04IS4F06 (EF629727)
Uncultured Chloroflexi bacterium clone W04IS5D12 (EF629743)
Uncultured Chloroflexi bacterium clone W04ISB2R (EF629750)
Uncultured Chloroflexi bacterium clone W04ISD4R (EF629755)
Uncultured bacterium clone TO10-919C25 (JX206630)
Uncultured bacterium clone TO10-97C21 (JX206596)
Uncultured bacterium clone TO10-919C18 (JX206624)
Uncultured bacterium clone AF10-3-7C14 (JN655226)
Uncultured bacterium clone TO10-922C19 (JX206654)
Uncultured bacterium clone TO10-97C26 (JX206601)
Uncultured bacterium clone PO10-3-1C11 (JN655330)
Uncultured bacterium clone TO10-97C27 (JX206602)
Uncultured bacterium clone PO10-3-1C15 (JN655334)
Uncultured bacterium clone PO10-3-7C16 (JN655362)
Uncultured bacterium clone AF10-97C25 (JX206498)
Uncultured bacterium clone TO10-922C30 (JX206663)
Uncultured Chloroflexi bacterium clone W04IS5C02 (EF629739)
Uncultured Chloroflexi bacterium clone W04ISB1R (EF629749)
Uncultured Chloroflexi bacterium clone W04IS5B07 (EF629736)
Uncultured Chloroflexi bacterium clone W04ISA5R (EF629762)
Uncultured Chloroflexi bacterium clone W04IS5B12 (EF629738)
Uncultured Chloroflexi bacterium clone W04ISB4R (EF629752)
Uncultured Chloroflexi bacterium clone W04ISE1R (EF629756)
Uncultured Chloroflexi bacterium clone W04IS4D08 (EF629720)
Uncultured Chloroflexi bacterium clone W04IS4B06 (EF629711)
Uncultured Chloroflexi bacterium clone W04ISD6R (EF629766)
Uncultured Chloroflexi bacterium clone W04IS5C5RF (EF629772)
Uncultured Chloroflexi bacterium clone W04ISD5R (EF629765)
Uncultured Chloroflexi bacterium clone W04IS5D01 (EF629742)
Uncultured Chloroflexi bacterium clone W04IS5C07 (EF629741)
Uncultured Chloroflexi bacterium clone W04IS4C02 (EF629713)
Uncultured Chloroflexi bacterium clone W04ISH2R (EF629761)
Uncultured Chloroflexi bacterium clone W04IS4A09 (EF629706)
Uncultured Chloroflexi bacterium clone W04IS5A05 (EF629733)
Uncultured Chloroflexi bacterium clone W04ISE4R (EF629757)
Uncultured Chloroflexi bacterium clone W04F1R (EF629758)
Uncultured Chloroflexi bacterium clone W04ISC6R (EF629764)
Uncultured Chloroflexi bacterium clone W04ISH5R (EF629770)
Uncultured Chloroflexi bacterium clone W04ISH6R (EF629771)
Uncultured Chloroflexi bacterium clone W04IS5E12 (EF629745)
Uncultured Chloroflexi bacterium clone W04ISH1R (EF629760)
Uncultured bacterium clone AF10-3-7C13 (JN655225)
Uncultured bacterium clone BA26-C2-seq (JX280320)
Uncultured bacterium clone TO10-97C12 (JX206588)
Uncultured bacterium clone TO10-919C28 (JX206633)
Uncultured bacterium clone TO10-922C15 (JX206651)
Anaerolineae
Uncultured Chloroflexi bacterium clone 9m05AISF08 (EF629773)
Uncultured bacterium clone IS-69 (GU982168)
Uncultured bacterium clone IS-47 (GU982149)
Uncultured bacterium clone IS-74 (GU982172)
Uncultured bacterium clone IS-63 (GU982163)
Uncultured bacterium clone IS-46 (GU982148)
Uncultured bacterium clone IS-66 (GU982166)
Uncultured bacterium clone IS-09 (GU982114)
Uncultured bacterium clone IS-23 (GU982126)
Uncultured bacterium clone IS-04 (GU982110)
Uncultured bacterium clone BA16-C24-seq (JX280280)
Uncultured bacterium clone BA16-C28-seq (JX280281)
Uncultured bacterium clone TV10-97C23 (JX206705)
Uncultured bacterium clone IS-53 (GU982155)
Caldilineae
Uncultured bacterium clone IS-60 (GU982161)
Uncultured bacterium clone PO10-3-1C24 (JN655343)
Uncultured bacterium clone TO10-97C10 (JX206586)
Uncultured bacterium clone TO10-97C32 (JX206607)
Uncultured bacterium clone TO10-919C5 (JX206612)
Uncultured bacterium clone IS-10 (GU982115)
Uncultured bacterium clone IS-44 (GU982146)
Uncultured Chloroflexi bacterium clone W04IS5A09 (EF629734)
Uncultured Chloroflexi bacterium clone W04IS5C04 (EF629740)
Uncultured Chloroflexi bacterium clone W04IS5E04 (EF629744)
Uncultured bacterium clone BA16-C54-seq (JX280283)
Uncultured bacterium clone BA26-C29-seq (JX280327)
Uncultured Chloroflexi bacterium clone W04IS4H09 (EF629731)
Uncultured bacterium clone IS-55 (GU982157)
Uncultured bacterium clone AF10-3-9C29 (JN655268)
Uncultured bacterium clone BA01-C3-seq (JX280169)
Uncultured bacterium clone BA100-C39-seq (JX280213)
Uncultured bacterium clone BA26-C37-seq (JX280333)
Uncertain affiliation
Uncultured bacterium clone TV10-97C4 (JX206696)
Uncultured bacterium clone TV10-912C6 (JX206718)
Uncultured bacterium clone BA26-C30-seq (JX280328)
Uncultured bacterium clone IS-83 (GU982180)
Uncultured bacterium clone BA26-C47-seq (JX280341)
Uncultured bacterium clone IS-84 (GU982181)
Uncultured bacterium clone IS-12 (GU982117)
Uncultured bacterium clone IS-64 (GU982164)
Uncultured bacterium clone IS-37 (GU982139)
Uncultured bacterium clone IS-40 (GU982142)
0.05

(g) Acidobacteria

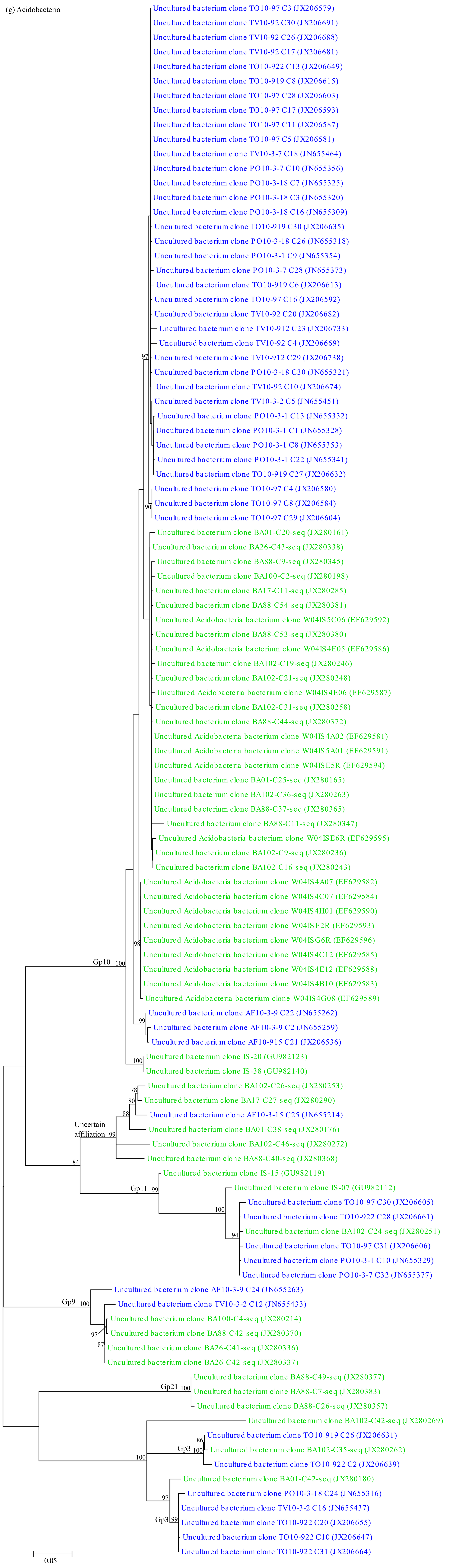

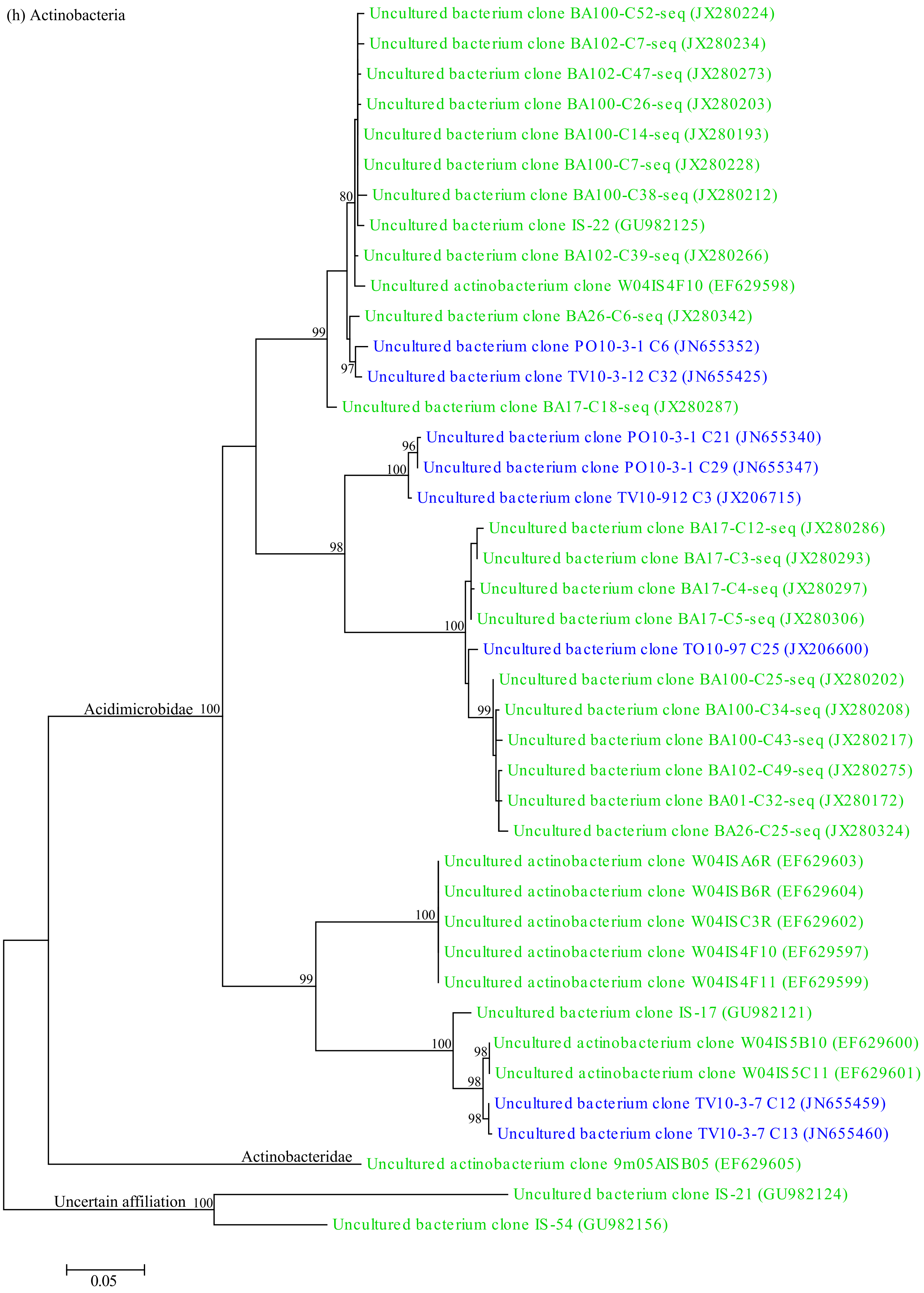
(h) Actinobacteria
Uncultured bacterium clone BA100-C52-seq (JX280224)
Uncultured bacterium clone BA102-C7-seq (JX280234)
Uncultured bacterium clone BA102-C47-seq (JX280273)
Uncultured bacterium clone BA100-C26-seq (JX280203)
Uncultured bacterium clone BA100-C14-seq (JX280193)
Uncultured bacterium clone BA100-C7-seq (JX280228)
80
Uncultured bacterium clone BA100-C38-seq (JX280212)
Uncultured bacterium clone IS-22 (GU982125)
Uncultured bacterium clone BA102-C39-seq (JX280266)
Uncultured actinobacterium clone W04IS4F10 (EF629598)
Uncultured bacterium clone BA26-C6-seq (JX280342)
99
Uncultured bacterium clone PO10-3-1 C6 (JN655352)
97
Uncultured bacterium clone TV10-3-12 C32 (JN655425)
Uncultured bacterium clone BA17-C18-seq (JX280287)
96
Uncultured bacterium clone PO10-3-1 C21 (JN655340)
100
Uncultured bacterium clone PO10-3-1 C29 (JN655347)
Uncultured bacterium clone TV10-912 C3 (JX206715)
Uncultured bacterium clone BA17-C12-seq (JX280286)
98
Uncultured bacterium clone BA17-C3-seq (JX280293)
Uncultured bacterium clone BA17-C4-seq (JX280297)
Uncultured bacterium clone BA17-C5-seq (JX280306)
100
Uncultured bacterium clone TO10-97 C25 (JX206600)
Uncultured bacterium clone BA100-C25-seq (JX280202)
Acidimicrobidae 100
99
Uncultured bacterium clone BA100-C34-seq (JX280208)
Uncultured bacterium clone BA100-C43-seq (JX280217)
Uncultured bacterium clone BA102-C49-seq (JX280275)
Uncultured bacterium clone BA01-C32-seq (JX280172)
Uncultured bacterium clone BA26-C25-seq (JX280324)
Uncultured actinobacterium clone W04ISA6R (EF629603)
Uncultured actinobacterium clone W04ISB6R (EF629604)
100
Uncultured actinobacterium clone W04ISC3R (EF629602)
Uncultured actinobacterium clone W04IS4F10 (EF629597)
99
Uncultured actinobacterium clone W04IS4F11 (EF629599)
Uncultured bacterium clone IS-17 (GU982121)
100
98
Uncultured actinobacterium clone W04IS5B10 (EF629600)
98
Uncultured actinobacterium clone W04IS5C11 (EF629601)
Uncultured bacterium clone TV10-3-7 C12 (JN655459)
98
Uncultured bacterium clone TV10-3-7 C13 (JN655460)
Actinobacteridae
Uncultured actinobacterium clone 9m05AISB05 (EF629605)
Uncertain affiliation 100
Uncultured bacterium clone IS-21 (GU982124)
Uncultured bacterium clone IS-54 (GU982156)
0.05

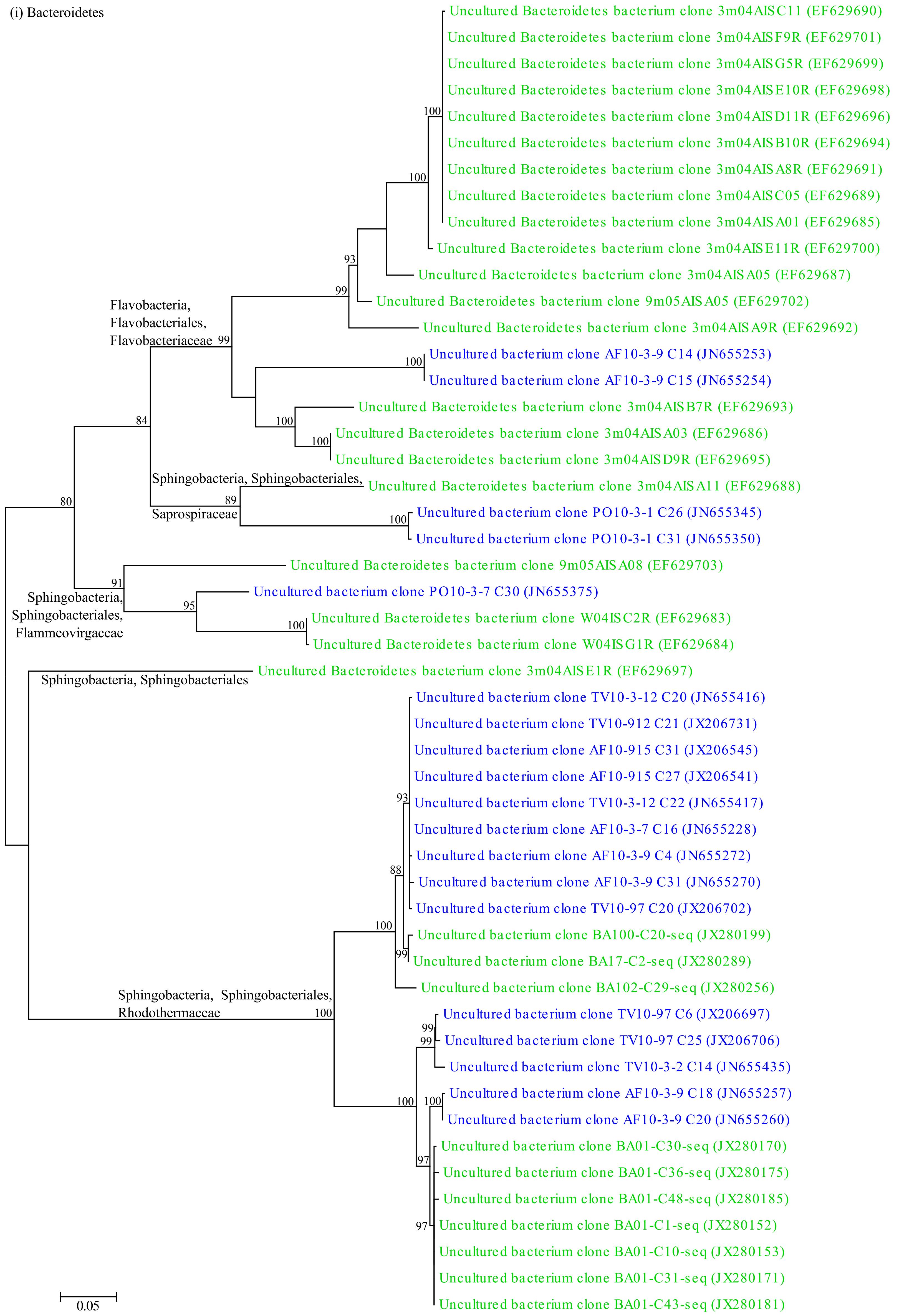
(i) Bacteroidetes
Uncultured Bacteroidetes bacterium clone 3m04AISC11 (EF629690)
Uncultured Bacteroidetes bacterium clone 3m04AISF9R (EF629701)
Uncultured Bacteroidetes bacterium clone 3m04AISG5R (EF629699)
Uncultured Bacteroidetes bacterium clone 3m04AISE10R (EF629698)
Uncultured Bacteroidetes bacterium clone 3m04AISD11R (EF629696)
Uncultured Bacteroidetes bacterium clone 3m04AISB10R (EF629694)
Uncultured Bacteroidetes bacterium clone 3m04AISA8R (EF629691)
Uncultured Bacteroidetes bacterium clone 3m04AISC05 (EF629689)
Uncultured Bacteroidetes bacterium clone 3m04AISA01 (EF629685)
Uncultured Bacteroidetes bacterium clone 3m04AISE11R (EF629700)
Uncultured Bacteroidetes bacterium clone 3m04AISA05 (EF629687)
Uncultured Bacteroidetes bacterium clone 9m05AISA05 (EF629702)
Uncultured Bacteroidetes bacterium clone 3m04AISA9R (EF629692)
Flavobacteria, Flavobacteriales, Flavobacteriaceae
Uncultured bacterium clone AF10-3-9 C14 (JN655253)
Uncultured bacterium clone AF10-3-9 C15 (JN655254)
Uncultured Bacteroidetes bacterium clone 3m04AISB7R (EF629693)
Uncultured Bacteroidetes bacterium clone 3m04AISA03 (EF629686)
Uncultured Bacteroidetes bacterium clone 3m04AISD9R (EF629695)
Sphingobacteria, Sphingobacteriales, Saprospiraceae
Uncultured Bacteroidetes bacterium clone 3m04AISA11 (EF629688)
Uncultured bacterium clone PO10-3-1 C26 (JN655345)
Uncultured bacterium clone PO10-3-1 C31 (JN655350)
Uncultured Bacteroidetes bacterium clone 9m05AISA08 (EF629703)
Sphingobacteria, Sphingobacteriales, Flammeovirgaceae
Uncultured bacterium clone PO10-3-7 C30 (JN655375)
Uncultured Bacteroidetes bacterium clone W04ISC2R (EF629683)
Uncultured Bacteroidetes bacterium clone W04ISG1R (EF629684)
Sphingobacteria, Sphingobacteriales
Uncultured Bacteroidetes bacterium clone 3m04AISE1R (EF629697)
Uncultured bacterium clone TV10-3-12 C20 (JN655416)
Uncultured bacterium clone TV10-912 C21 (JX206731)
Uncultured bacterium clone AF10-915 C31 (JX206545)
Uncultured bacterium clone AF10-915 C27 (JX206541)
Uncultured bacterium clone TV10-3-12 C22 (JN655417)
Uncultured bacterium clone AF10-3-7 C16 (JN655228)
Uncultured bacterium clone AF10-3-9 C4 (JN655272)
Uncultured bacterium clone AF10-3-9 C31 (JN655270)
Uncultured bacterium clone TV10-97 C20 (JX206702)
Uncultured bacterium clone BA100-C20-seq (JX280199)
Uncultured bacterium clone BA17-C2-seq (JX280289)
Uncultured bacterium clone BA102-C29-seq (JX280256)
Sphingobacteria, Sphingobacteriales, Rhodothermaceae
Uncultured bacterium clone TV10-97 C6 (JX206697)
Uncultured bacterium clone TV10-97 C25 (JX206706)
Uncultured bacterium clone TV10-3-2 C14 (JN655435)
Uncultured bacterium clone AF10-3-9 C18 (JN655257)
Uncultured bacterium clone AF10-3-9 C20 (JN655260)
Uncultured bacterium clone BA01-C30-seq (JX280170)
Uncultured bacterium clone BA01-C36-seq (JX280175)
Uncultured bacterium clone BA01-C48-seq (JX280185)
Uncultured bacterium clone BA01-C1-seq (JX280152)
Uncultured bacterium clone BA01-C10-seq (JX280153)
Uncultured bacterium clone BA01-C31-seq (JX280171)
Uncultured bacterium clone BA01-C43-seq (JX280181)
0.05
